# Small Molecule Drugs That Inhibit Phagocytosis

**DOI:** 10.3390/molecules28020757

**Published:** 2023-01-12

**Authors:** Melika Loriamini, Melissa M. Lewis-Bakker, Kayluz Frias Boligan, Siming Wang, Mairead B. Holton, Lakshmi P. Kotra, Donald R. Branch

**Affiliations:** 1 Centre for Innovation, Canadian Blood Services, Toronto, ON M5G 2M1, Canada; 2 Department of Laboratory Medicine and Pathobiology, University of Toronto, Toronto, ON M5S 1A8, Canada; 3 Krembil Research Institute, University Health Network, Toronto, ON M5G 1L7, Canada; 4 Department of Pharmaceutical Sciences, Leslie Dan Faculty of Pharmacy, University of Toronto, Toronto, ON M5S 3M2, Canada; 5 Department of Medicine, University of Toronto, Toronto, ON M5S 1A8, Canada; 6 Keenan Research Centre, Canadian Blood Services, Toronto, ON M5B 1W8, Canada

**Keywords:** immune cytopenias, phagocytosis inhibitors, drug discovery, medicinal chemistry

## Abstract

In our initial publication on the in vitro testing of more than 200 compounds, we demonstrated that small molecules can inhibit phagocytosis. We therefore theorized that a small molecule drug discovery-based approach to the treatment of immune cytopenias (ITP, AIHA, HTR, DHTR) is feasible. Those earlier studies showed that small molecules with anti-phagocytic groups, such as the pyrazole core, are good models for producing efficacious phagocytosis inhibitors with low toxicity. We recently screened a chemical library of 80 compounds containing pyrazole/isoxazole/pyrrole core structures and found four hit molecules for further follow-up, all having the pyrazole core structure. Subsequent evaluation via MTT viability, LDH release, and apoptosis, led to the selection of two lead compounds with negligible toxicity and high efficacy. In an in vitro assay for inhibition of phagocytosis, their IC_50_ values were 2–4 µM. The rational development of these discoveries from hit to lead molecule stage, viz. independent synthesis/scale up of hit molecules, and in vivo activities in mouse models of autoimmune disease, will result in the selection of a lead compound(s) for further pre-clinical evaluation.

## 1. Introduction

Immune cytopenias are conditions in which people generate antibodies against certain types of hematopoietic cells in their blood [1,2,3]. Cells become coated with antibodies under these circumstances and are identified by the Fcγ receptors (FcγR) on the membrane of mononuclear phagocytes. Such recognition by monocyte-macrophages results in extravascular hemolysis in the spleen and/or liver macrophages due to FcγR-mediated phagocytosis [2]. Affected individuals can face severe and sometimes even life-threatening complications due to this process [2]. Immune cytopenias have many categories [2], including (i) immune thrombocytopenia (ITP; autoimmune disease characterized by increased platelet destruction in the spleen and liver and/or decreased platelet production in the bone marrow); (ii) hemolytic disease of the fetus and newborn (HDFN; maternal hemolytic antibodies crossing the placenta); (iii) autoimmune hemolytic anemia (AIHA; phagocytosis of autoantibody-coated red blood cells); (iv) alloimmune hemolytic anemias such as hemolytic transfusion reaction (HTR; phagocytosis of donor red blood cells due to preformed hemolytic alloantibodies to the donor red cell antigens); (v) delayed hemolytic transfusion reaction (DHTR; development of hemolytic alloantibodies following transfusion); and (vi) autoimmune neutropenia (AIN) associated with autoantibodies produced against neutrophils, mainly affecting children.

The common factor of all immune cytopenias is the destruction of particular blood cells opsonized with antibodies, which requires FcγR-mediated phagocytosis. Thus, developing small molecule agents (drugs) that provides blockade of the phagocytosis would ameliorate the various immune cytopenias, and elucidation of such drugs would provide a useful clinical intervention [3,4].

The chemical synthesis and evaluation of several small molecules as potential therapeutics for ITP have been previously reported [5,6]. These heterocyclic molecules consisted of substituted pyrroles, pyrazoles, and bis-pyrazoles with S-S, C-C and C-N linkages. Evaluation of these molecules was conducted using the monocyte monolayer assay (MMA), a highly adaptable in vitro assay that can be modified to examine different aspects of antibody and Fc receptor (FcR)-mediated phagocytosis in both research and clinical settings [7,8]. Many FcRs (such as FcRI, FcRIIA, FcRIIB, FcRIIC, and FcRIIIA) are important in immunological responses and are expressed on these monocytes and macrophages. Antibody-sensitized red blood cells (RBCs) attach to and/or activate FcRs, prompting the monocyte-macrophages to phagocytose them, a mechanism the MMA uses to its advantage [7,8,9]. Adherent monocytes from peripheral blood mononuclear cells isolated from whole blood in mammals are used for testing, and the number of phagocytosed anti-D-opsonized red cells is visually enumerated [7]. It was demonstrated that these small molecules were capable of inhibiting phagocytosis [5,6], although robust inhibition was not observed.

In a continued effort to identify in vitro phagocytosis inhibitor(s) with high efficacy and negligible toxicity, and subsequently move to in vivo testing in animal models of immune cytopenias, molecules with additional structural modification(s) were examined. Here, in this report, are results of an in vitro screen of 80 compounds—obtained from a commercially available chemical library—leading to two compounds with negligible toxicity and high efficacy for inhibition of phagocytosis. These two molecules fulfill criteria as potential lead compounds and can be advanced to the evaluation in animal models of autoimmune cytopenias.

## 2. Results

### 2.1. Selection of Compounds for In Vitro Screening

**Figure 1:**
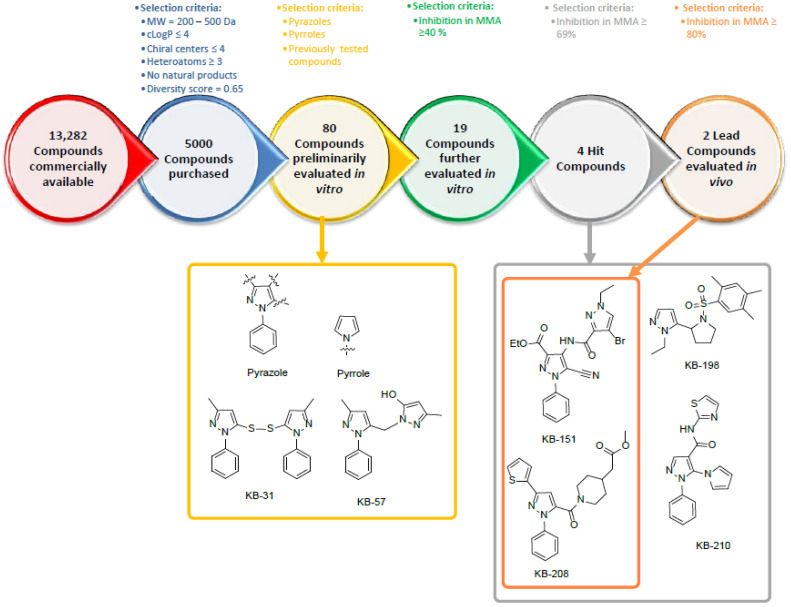
Selection process of compounds screened in vitro for the inhibition of phagocytosis.

Figure 1 shows the selection process of small molecule agents for the inhibition of phagocytosis. An in silico screen of a commercial library containing more than 13,000 compounds was conducted using physicochemical parameters. From this screen, a 5000 compound library was acquired and further narrowed to 80 compounds, using several core structural moieties of previously investigated small molecules [6]. These 80 compounds were evaluated in vitro, resulting in two lead compounds, as described in subsequent sections below.

### 2.2. Monocyte Monolayer Assay (MMA)

**Figure 2:**
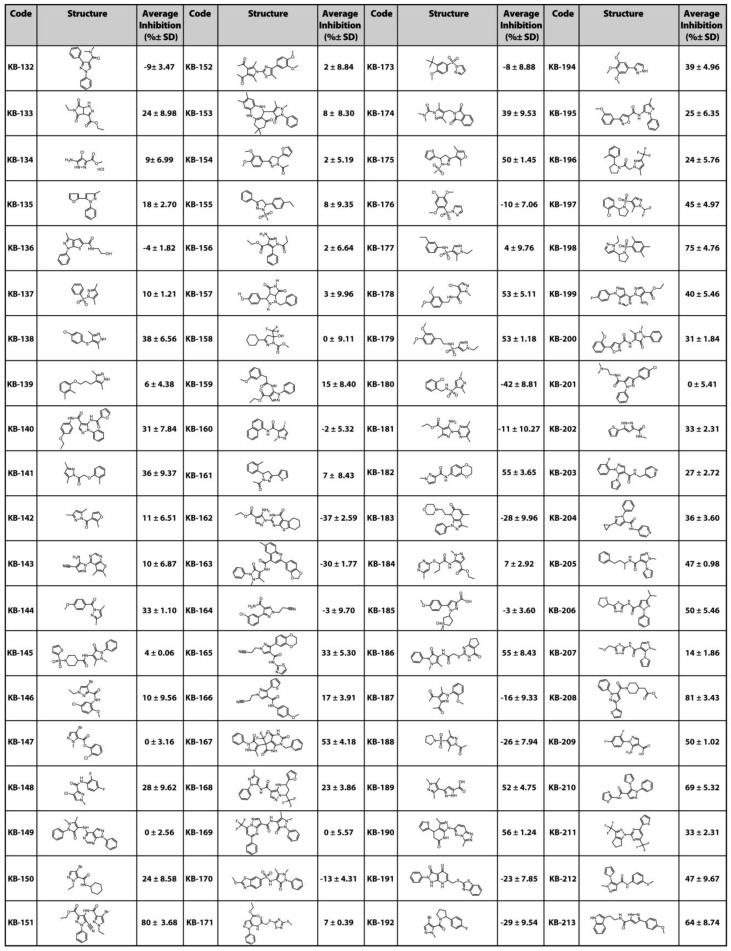
Chemical structures and % inhibition (mean ± SD) in MMA of the 80 compounds preliminarily evaluated in vitro. Compounds were screened at 5 µM and compared to the reference compound, intravenous immunoglobulin (IVIG) at 1 mg/mL.

**Figure 3:**
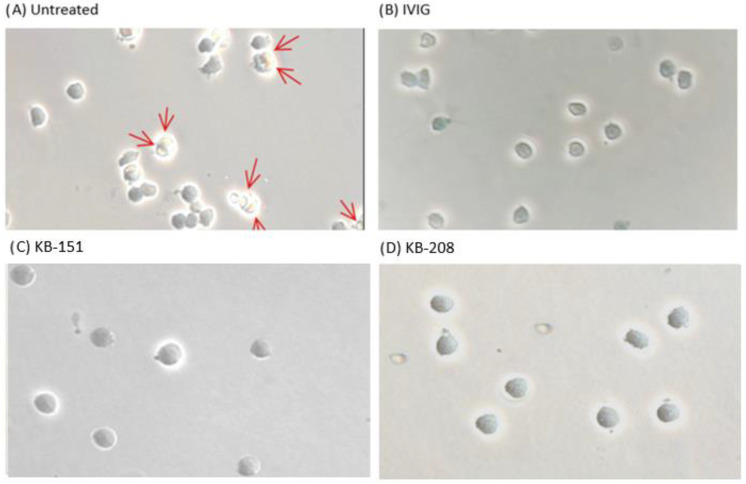
Phase contrast microscopy images. (**A**) Untreated human monocytes- representing monocytes phagocytosing RBCs opsonized with anti-D. Red arrows indicate RBCs that are phagocytosed (RBCs inside of monocytes). (**B**) Inhibitor control- Human monocytes treated with 1 mg/mL of IVIG. (**C**) Human monocytes treated with 100 µM KB-151. (**D**) Human monocytes treated with 100 µM KB-208.

The selected 80 compounds were initially screened in an in vitro phagocytosis experiment, referred to as the monocyte monolayer assay (MMA) [7,8,9], at a concentration of 5 µM (Figure 2). Compounds were compared to the reference compound, intravenous immunoglobulin (IVIG) at a concentration of 1 mg/mL. Following this preliminary screen, 19 compounds that inhibited anti-D-opsonized Rh(D+) red cell phagocytosis by at least 40% were identified and chosen for further evaluation (Table 1). Phase contrast microscopy images of the MMA show phagocytosis of untreated red blood cells (RBCs) by human monocytes (Figure 3A) and the inhibition in RBCs treated with either IVIG or compounds (Figure 3B–D).

### 2.3. LDH Release and MTT Assays

**Figure 4:**
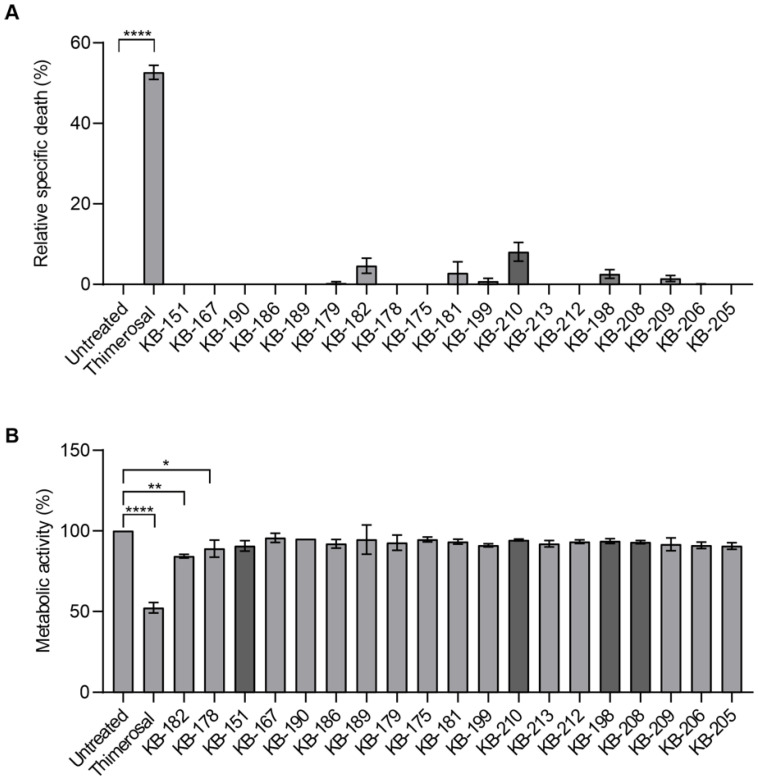
(**A**). LDH results: *Y*-axis shows the percentage of cell death. Thimerosal at 100 µM (**** *p* ≤ 0.0001) shows a significant difference compared to untreated control. (**B**). MTT results: *Y*-axis shows the percentage of cell viability which relates to the cell metabolic activity. Thimerosal at 100 µM (**** *p* ≤ 0.0001), **KB-178** (* *p* < 0.05) and **KB-182** (** *p* < 0.005) show significant differences compared to untreated control. Bars represent the mean ± SD and N = 3. The four hit compounds are shown in a darker colour.

Additionally, these 19 compounds were tested for toxicity at a concentration of 5 µM, using an LDH release assay (Figure 4A) for a weakened cell membrane and the MTT assay for cell viability (Figure 4B). Both are colorimetric assays, but LDH relies on the release of LDH enzymes into the culture medium after cell membrane disruption [10]. As a result, the production of color suggests cytolysis. Thimerosal is a potent inhibitor of anti-Rh(D)-coated red blood cells, but does display moderate toxicity, therefore, 100 µM was used as a positive control for cell damage [11]. Compounds **KB-181**, **KB-182**, **KB-198**, **KB-199**, **KB-209**, and **KB-210** demonstrated little toxicity in these experiments compared to the control, thimerosal (100 µM), but were not significant when compared to untreated control. None of the other compounds released any LDH enzymes into the culture medium (Figure 4A).

The MTT test is a metabolic activity assay based on the enzymatic conversion of MTT in mitochondria, and color formation is an indicator of cell viability [12,13]. Thimerosal at 100 µM (**** *p* ≤ 0.0001), **KB-178** (* *p* < 0.05), and **KB-182** (** *p* < 0.005) all demonstrated statistically significant changes when compared to the untreated control (tested by Kruskal–Wallis test). Other compounds showed no statistically significant changes from the untreated control (Figure 4B).

### 2.4. Dose-Dependent Inhibition

**Figure 5:**
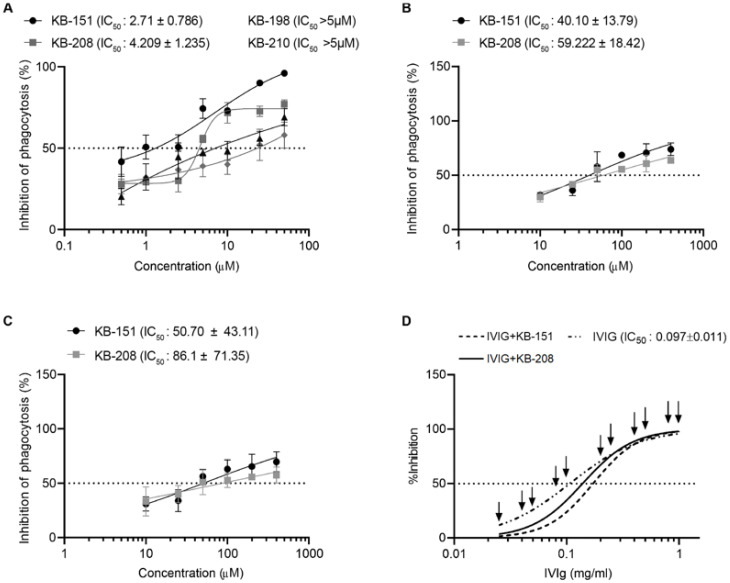
(**A**) Human PBMC’s IC_50_s for the top four compounds. (**B**) Mouse PBMC’s IC_50_s for the two lead compounds. (**C**) Mouse macrophages, RAW 264.7′s, IC_50_s for the two lead compounds. (**D**) Dose-inhibitory response of IVIG alone (IC_50_ = 0.097 ± 0.011) or with addition of **KB-208** (___) or **KB-151** (----) at their IC_50_ (hPBMCs) concentrations (4µM and 3µM, respectively). Lead compounds are administered along with IVIG at each IVIG concentration used in its titration (arrows). Bars represent mean ± SD, and N = 3.

For further investigation, compounds that inhibited phagocytosis by at least 65 percent in the primary screening and those that showed no toxicity in the LDH and MTT assays were selected. The selected compounds were **KB-151**, **KB-198**, **KB-208**, and **KB-210**. The dose-inhibitory responses of these four drugs were determined, and the IC_50_ values were calculated. IC_50_ values for **KB-198** and **KB-210** were more than 5 µM; IC_50_ values for **KB-151** and **KB-208** were 2.7 ± 0.8 µM and 4.2 ± 1.2 µM, respectively (Figure 5A). Ten different donors were tested with **KB-151** to determine the mean ± SD of the IC_50_. Although similar to the original value, as expected, the value for the IC_50_ increased to 8.7 ± 9.2 µM. Similar additional testing for the IC_50_ using 10 different donors with **KB-208** revealed IC_50_ 10.1 ± 7.9 µM. Since the dose-response curves and IC_50_ values for **KB-151** and **KB-208** were the most favorable, and their MTT and LDH results revealed no significant toxicity, they were chosen for further evaluation as lead compounds. The IC_50_ values in mice PBMCs (BALB/c) and RAW 264.7 (mouse macrophage cell line) for the lead compounds (Figure 5B,C, respectively) were also calculated. The IC_50_ values for **KB-151** were 40.1 ± 13.8 µM (mouse PBMCs) and 50.7 ± 43.1 µM (RAW 264.7); IC_50_ values for **KB-208** were 59.2 ± 18.4 µM (mouse PBMCs) and 86.1 ± 71.6 µM (RAW 264.7).

The cooperation of lead compounds **KB-151** and **KB-208** with IVIG in a dose-inhibitory assay were also evaluated. IVIG was titrated alone and in combination with each test compound (IVIG + test compound), at each IVIG concentration using their IC_50_ concentrations (**KB-151**, 3 µM and **KB-208**, 4 µM). Shifting of the IVIG titration curve would indicate cooperation (synergy) with the KB compound(s), however, the compounds had no effect on the IVIG dose-response curve (Figure 5D).

### 2.5. Additional Toxicity Testing

**Figure 6:**
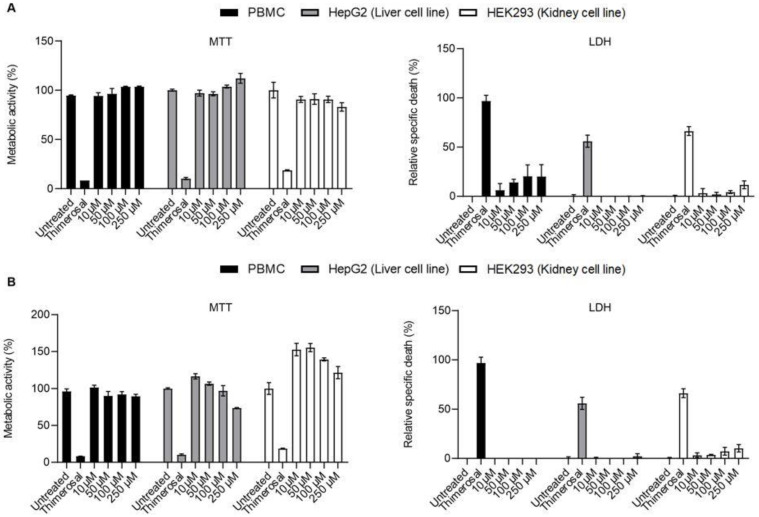
MTT (**left**) and LDH (**right**) results for PBMC (Black), Hep G2 (Grey), and HEK-293 (White) using high concentration (up to 250 µM) of **KB-151** (**A**) and **KB-208** (**B**). *Y*-axis for MTT graph shows the percentage of metabolic activity which is related to the cell viability, and for LDH graph, it shows the percentage of relative specific death which related to the cell toxicity. *X*-axis of both MT and LDH show different concentrations of **KB-151** and **KB-208**, Thimerosal at 100 µM as control toxicity (**** *p* ≤ 0.0001), and untreated control. Bars represent mean ± SD, and N = 3.

**Figure 7:**
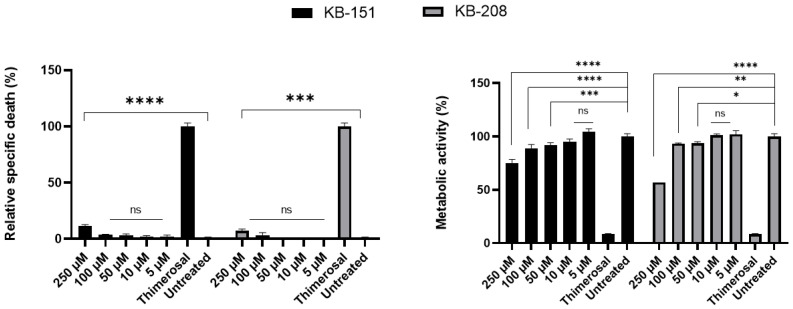
Toxicity assay after 24 h incubation of PBMCs with **KB-151** and **KB-208**. MTT (**right**) and LDH (**left**) results for PBMC using high concentration (up to 250 µM) of **KB-151** (black) and **KB-208** (grey). *Y*-axis for MTT graph shows the percentage of metabolic activity which is related to the cell viability and for LDH graph, shows the percentage of relative specific death which related to the cell toxicity. *X*-axis of both MTT and LDH show different concentrations of **KB-151** and **KB-208**, Thimerosal at 100 µM as control toxicity (* *p* < 0.05, ** *p* < 0.01, *** *p* < 0.001 and **** *p* < 0.0001), and untreated control. Bars represent mean ± SD, and N = 3.

Further toxicity studies were conducted using higher concentrations of each lead compound **KB-151** and **KB-208**—up to 250 µM—in LDH, MTT, and apoptosis assays (Annexin V/PI). Figure 6 shows MTT and LDH results for PBMC, Hep G2, and HEK-293 using high concentration (up to 250 µM) of **KB-151** (Figure 6A) and **KB-208** (Figure 6B). Similar experiments with PBMCs incubated with different concentration of **KB-151** and **KB-208** for 24 h were also performed (Figure 7). All compounds showed low to no toxicity using peripheral blood mononuclear cells (PBMCs), liver Hep G2, and kidney HEK293 cell. No significant difference was found between cells treated with leading compound and untreated control. PBMCs showed more than 90% metabolic activity up to 100 µM and less than 11% LDH released in culture media at 250 µM after 24 h incubations.

### 2.6. Apoptosis Assays

**Figure 8:**
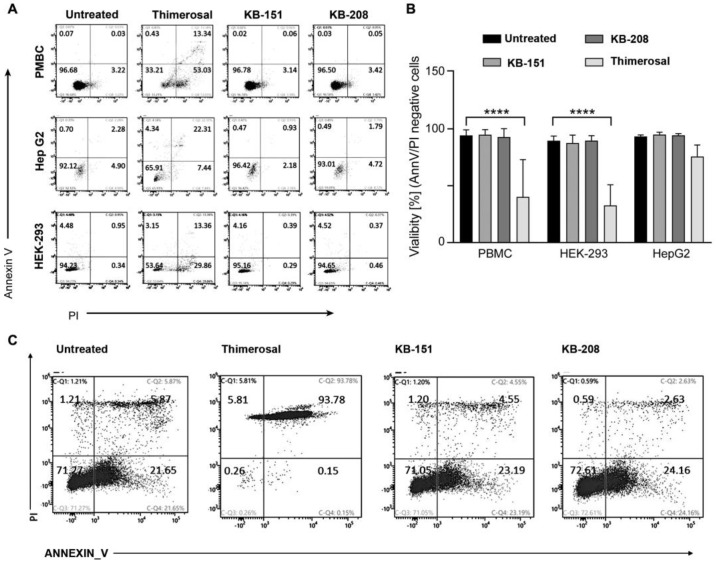
Apoptosis assay for PBMC, Hep G2, and HEK-293 all treated with **KB-151** and **KB-208** at 250 µM concentration for 1 h. (**A**) Representative example of treatments (**B**) Summary of three independent experiments (**** *p* < 0.0001). (**C**) PBMC treated with **KB-151** and **KB-208** at 250 µM concentration for 24 h.

Apoptosis assay results for PBMC, Hep G2, and HEK-293 are represented in Figure 8. **KB-151** and **KB-208** showed the same patterns in PBMC, Hep G2, HEK-293, and were similar to untreated cells (Figure 8A). They also showed no significant changes in % viability compared to untreated control in the different cell types (Figure 8B). Similar experiments with PBMCs incubated with different concentrations of **KB-151** and **KB-208** for 24 h were also performed (Figure 8C). In the apoptosis assay, only 70% of the cells were viable, in comparison to 60% for MTT and >89% by LDH.

## 3. Discussion

There are few proven treatments for severe cases of immune cytopenia. Most novel therapies have targeted ITP over other variants of immune cytopenias, such as AIHA, HTR, DHTR, or HDFN. Prevalent therapies include corticosteroids (dexamethasone, prednisone) and rituximab (anti-CD20), as well as IVIG and anti-D [14,15,16]. There are various alternatives to the use of IVIG in ITP, including thrombopoietin receptor agonists (TPOS-Ras; Eltrombopag and Avatrombopag), anti-D, spleen tyrosine kinase (Syk) inhibitors (fostamatinib), and splenectomy [17,18,19,20]. However, since current therapies requires time to be effective, they cannot be used in acute ITP, HTR, DHTR, or HDFN and fulminant AIHA, where the patient may rapidly hemolyze to death. As a result, treatments that can rapidly reverse the immune-mediated destruction of specific blood cells would be highly beneficial. More specifically, they would provide additional time for implementing other therapeutic strategies leading to a higher chance of patient survival.

To date, only small molecules have been developed to treat ITP, such as the orally administered Syk inhibitor Fostamatinib, for patients with chronic ITP who are unresponsive to other treatment options [17,18]. This latest ATP-competitive prodrug is rapidly dephosphorylated to its active metabolite R-406 in the stomach and acts by blocking Fc-activating receptors. Syk-inhibitors are not specific for monocyte-macrophage Fc receptors, but also target critical signaling pathways in B-cells and T-cells. Other FDA-approved small molecules currently used in the treatment of ITP include the orally administered TPO-RAs Eltrombopag and Avatrombopag [19]. Although their mechanism of action involves stimulation of the TPO receptor on megakaryocytes and hematopoietic pluripotent stem cells, these TPO-RAs are efficacious because they promote platelet precursor survival and increase platelet production by reversing the low platelet production in the bone marrow. Unfortunately, treatments with current small molecules are limited to chronic ITP patients and may require long-term use. Hence, developing small molecules that would be more specific, orally bioavailable, and have a broader application is a worthwhile endeavour. These inhibitors could be administered as sole therapeutics or co-administered with other ITP treatment options. Interest in drug discovery to identify small molecule phagocytosis inhibitors led to our initial publication showing compounds with a disulfide bond (S-S) as in vitro inhibitors of phagocytosis [5]. Since reactive groups such S-S are undesirable in good druggable compounds, additional focused libraries based on 2nd generation phenyl pyrazoles and with desirable chemical structures were synthesized [5]. An iterative exploration of 3rd generation structures incorporating pyrazoles, isoxazoles (for solubility improvement), and pyrroles linked to various heterocycles via alkyl groups (for favourable interactions with the reactive portions in the cell surface of the macrophage) (unpublished results; Figure S1 Supplementary Material) was also conducted. These modified structures were less superior at blocking in vitro phagocytosis, with % inhibition less than 40% (unpublished results). Attempts were made to build computational models using 2D quantitative structure-activity relationship (2D QDSAR), but reliable models could not be obtained.

As reported herein, a focused effort involving in silico screening of a commercial library of over 13,000 compounds was conducted, and a subset of 5000 compounds was obtained. The compounds in the subset were chosen using criteria such as the number of chiral centers (less than 4) and no natural products. Additionally, since one of the goals of this research is to subsequently move to in vivo testing in animal models of immune cytopenias, drug-like properties such as cLogP (no greater than 4), heteroatoms (greater than 3), and molecular weight (200–500 Da) were also considered. This library was further filtered for compounds with similar structural characteristics of previous efficacious small molecules, such as substituted phenyl pyrazole or pyrrole, or the structural features of KB-57—an active compound reported in our previous publication [5] with some in vivo activity. From this filter, a focused library of 80 diverse compounds, with predictable solubility and small molecule drug-like characteristics, was obtained (Figure 1 and Figure 2). Of the 80 compounds evaluated, **KB-151** and **KB-208** significantly inhibited phagocytosis in human monocyte-macrophages, at low µM IC_50_ concentrations. The use of 10 donors in the phagocytosis assays of each hit compound allowed for better determination of mean IC_50_ (±SD) values. Inevitably, the donor-to-donor variability subsequently led to variability in the observed IC_50_ values. Nevertheless, the observed IC_50_ values are considered low and relatively reproducible.

Both lead compounds **KB-151** and **KB-208** exhibited minimal to no toxicity in vitro up to 250 µM (highest concentration tested) at 1 h, but after 24 h at 250 μM, toxicity was observed. These toxicity results are promising and should pose no concerns, since the compounds would likely not be administered at such high doses once moved to in vivo experiments.

**KB-151** and **KB-208** were also able to inhibit phagocytosis in a murine system, albeit at a higher IC_50_ values. There are considerable differences between human and mouse monocyte-macrophages [21], therefore, the observed differences in IC_50_ values were not surprising. However, these IC_50_ values in mouse monocytes and macrophages indicate the starting doses of these compounds that should be used when they are evaluated in vivo, in the mouse model of ITP.

The pyrazole-containing compounds described herein show promise as potential small molecule inhibitors of phagocytosis. However, improved efficacy of these compounds, including the hit molecules, needs to be investigated in SAR studies, through modification or elimination of functional groups on the pyrazole core moiety. Future in vivo evaluation of these lead compounds may include (i) efficacy to ameliorate experimental ITP and AIHA; (ii) optimal administration route (IP, IV, subcutaneous, peroral) and (iii) dosing, with and without low-dose IVIG (for synergy). The lead candidate(s) that shows efficacy to reverse ITP and/or AIHA, or cooperate with IVIG to increase efficacy, may be tested for toxicity by pathological exams, histology, and immunohistochemistry of multiple organs. Likewise, further experiments are needed to determine the mechanism of action, since the possibility exists for blockade of Fc receptor proteins by these small molecules. Still, this effect is difficult to prove and, alternatively, they may affect signaling pathways. Additionally, the pharmacokinetics of the efficacious molecules, as well as the biodistribution of the molecules once administered, may be examined.

## 4. Materials and Methods

### 4.1. Selection of Compounds for Preliminary In Vitro Screening

From a commercial library of over 13,000 compounds, an in silico screen was conducted using physicochemical parameters, such as the number of chiral centers, heteroatoms, molecular weight and cLogP values. From these results, a 5000 compound library was acquired in the form of 10 mM DMSO solutions. Using several core structural moieties of previously investigated small molecules, this 5000 compound library was further narrowed to 80 compounds for preliminary in vitro iterative exploration.

The structures and purity for all 5000 compounds, including the final 80 selected and tested compounds, were recorded using ^1^H-NMR spectroscopy and/or HPLC-ELSD-DAD-MS. These data were supplied by the chemical library vendor and verified by us. All compounds are at least 95% pure, as confirmed by the chemical library vendor.

The four hit compounds (KB-151, KB-198, KB-208, and KB-210) were further analyzed in our laboratories using ^1^H-NMR, HPLC-PDA-MS, and UHPLC-HRMS/MS, and their purities were re-evaluated.

### 4.2. Characterization by ^1^H-NMR

^1^H-NMR experiments were recorded on a Bruker Avance 400 MHz spectrometer and chemical shifts are reported in δ ppm using CDCl_3_ (with tetramethylsilane as the internal standard) (Supplementary Data Figures S3–S6).

### 4.3. Characterization by UHPLC-HRMS/MS

The UHPLC-HRMS/MS profiles of the compounds (Supplementary Data Figures S7–S10) were obtained by a tandem LC-MS/MS system. UHPLC was performed on a Thermo Scientific Ultimate 3000 UHPLC system with a Thermo Scientific Hypersil Gold C_18_ column (50 mm × 2.1 mm, 1.9 µm I.D.) equipped with guard column. The mobile phase for UHPLC analysis consisted of water (A) and acetonitrile (B), each with 0.1% formic acid. The sample injection plate and the column were maintained at 5 °C and 40 °C, respectively. The gradient was programmed as follows: 0–0.5 min (50% A/50% B), 0.5–2 min (50% A/50% B to 0% A/100% B, gradient), 2–10 min (0% A/100% B), 10–10.5 min (0% A/100% B to 50% A/50% B, gradient), and 10.5–15 min (50% A/50% B). The flow-rate was 0.4 mL/min.

HRMS/MS was performed on a Thermo Scientific Q Exactive mass spectrometer equipped with a HESI-II probe. MS data were acquired in positive polarity, and the full MS (MS1) data (300–500 Da; resolution = 140,000) and parallel reaction monitoring (MS2) data (resolution = 17,500) were collected.

### 4.4. % Purity and Characterization by PDA

The purity of the compounds (two solvent systems; Supplementary Data Table S1 and Figures S11–S17) was determined by tandem HPLC-PDA-MS equipped with a Waters^®^ 2545 binary gradient module, Waters^®^ PDA 2998 photodiode array detector (190–800 nm) and a Waters^®^ QDa mass spectrometer (200–1200 Da). HPLC was performed on an X-Bridge^®^ analytical C_18_ column (4.6 mm × 150 mm, 5 µm I.D.). Mass spectra were recorded using ESI (+ve) mode (200–800 Da). All HPLC solvents are HPLC grade. The purity methods used for analysis are as follows:

Method A: The mobile phase for HPLC-PDA-MS analysis consisted of water (A) and methanol (B), each with 0.1% formic acid. The gradient was programmed as follows: 0–9 min (50% A/50% B to 2% A/98% B, gradient), 9–10 min (2% A/98%B); 10–11 min (2% A/98% B to 50% A/50%B, gradient), and 11–15 min (50% A/50%B). The flow-rate was 1 mL/min.

Method B: The mobile phase for analysis consisted of water (A) and acetonitrile (B), each with 0.1% formic acid. The gradient was programmed as follows: 0–9 min (50% A/50% B to 2%A/98% B, gradient), 9–10 min (2% A/98%B); 10–11 min (2% A/98% B to 50% A/50%B, gradient), and 11–15 min (50% A/50%B). The flow-rate was 1 mL/min.

### 4.5. Spectroscopic Data

**KB-151**: ^1^H-NMR (CDCl_3_, 400 MHz) δ ppm: 10.42 (s, 2H, H6), 7.73–7.70 (m, 2H, H1), 7.57–7.48 (m, 4H, H2 & H3), 4.52 (q, *J* = 7.3 Hz, 2H, H4), 4.26 (q, *J* = 7.5 Hz, 2H, H8), 1.57 (t, 3H, H5), 1.47 (t, 3H, H9); HRMS (HESI) *m/z*: [M+H]^+^ Calcd for C_19_H_18_BrN_6_O_3_ 457.0618; found 457.0623.

**KB-198**: ^1^H-NMR (CDCl_3_, 400 MHz) δ ppm: 7.50 (s, 1H, H13), 7.22 (d, *J* = 2.0 Hz, 2H, H3), 6.95 (s, 1H, H10), 5.90 (d, 1H, H4), 5.07 (d, *J* = 4.2, 7.8 Hz, 1H, H5), 4.19–4.02 (m, 2H, H2), 3.64 (dt, *J* = 7.2, 10 Hz, 1H. H8′), 3.44–3.39 (m, 1H, H8″), 2.42 (s, 3H, H9), 2.35–2.26 (m, 1H, H6′), 2.21 (s, 3H, H12), 2.18 (s, 3H, H11), 2.12–1.95 (m, 2H, H6″ & H7′), 1.92–1.85 (m, 1H, H7″), 1.38 (t, 3H, H1); HRMS (HESI) *m/z*: [M+H]^+^ Calcd for C_18_H_26_N_3_O_2_S 348.1740; found 348.1739.

**KB-208**: ^1^H-NMR (CDCl_3_, 400 MHz) δ ppm: 7.43–7.35 (m, 5H, H1, H2 & H3), 7.29 (d, *J* = 4.8 Hz, 1H, H4), 7.95 (dd, *J* = 3.7, 5.0 Hz, 1H, H5), 6.91 (s, 1H, H7), 6.84 (d, 1H, H6), 4.77 (t, *J* = 15.3 Hz, 2H, H8′), 3.67 (s, 3H, H12), 3.17 (t, *J* = 13.9 Hz, 1H, H8^a^″), 2.80 (t, 1H, H8^b^″), 2.27 (dd, *J* = 2.6, 7.5 Hz, 2H, H11), 2.15–2.06 (m, 1H, H10), 1.81 (dd, *J* = 14.1, 25.0 Hz, 2H, H9′), 1.37–1.24 (m, 2H, H9″); HRMS (HESI) *m/z*: [M+H]^+^ Calcd for C_22_H_24_N_3_O_3_S 410.1533; found 410.1531.

**KB-210**: ^1^H-NMR (CDCl_3_, 400 MHz) δ ppm: 8.33 (s, 1H, H4), 7.40 (d, *J* = 3.5 Hz, 1H, H8), 7.38–7.34 (m, 3H, H1 & H2′), 7.20–7.15 (m, 2H, H2″ & H3), 6.96 (d, 1H, H6, H7), 6.83–6.82 (m, 2H, H5), 6.53–6.52 (m, 2H, H6); HRMS (HESI) *m/z*: [M+H]^+^ Calcd for C_17_H_14_N_5_OS 336.0914; found 336.0912.

### 4.6. Primary Cells and Cell Lines

Upon informed consent, blood from healthy volunteers was collected in ac-id-citrate-dextrose (ACD) anticoagulant-containing tubes. Mononuclear cells (PBMC) were obtained by density centrifugation using Ficoll–Hypaque solution (Biochrom AG, Berlin Germany). Primary monocytes for functional phagocytosis assays were separated as described below.

All cell lines were purchased from American Type Culture Collection (ATCC, Manassas, VA, USA) and were tested free for mycoplasma. The cell lines used include: human 293[HEK-293, cat #CFL-1573, a human kidney embryo-derived cell line (kidney); human Hep G2[HEPG2, cat #HB-8065, a human-derived hepatocellular carcincoma cell line (liver); and LRRK2 parental RAW 264.7[RAW 264.7] mouse macrophages, cat #SC-6003. Hep G2 and HEK293 cell lines were cultured in Eagle’s Minimum Essential Medium (EMEM) supplemented with 10% fetal bovine serum, whereas RAW 264.7 cell lines were cultured in Dulbecco’s Modified Eagle Medium (DMEM) supplemented with 10% fetal bovine serum.

### 4.7. Phagocytosis Assay

The MMA to screen compounds for their ability to inhibit phagocytosis was performed as previously described [14,15]. Briefly, PBMCs were layered onto chamber slides and monocytes purified by adherence after 1 hr at 37 °C with 5% CO_2_. Drugs were solubilized in 100% DMSO and then diluted in RPMI to the final concentration of 5 µM, as previously reported [7]. Corresponding DMSO volumes in RPMI were used as a control. Final DMSO concentration used was ≤1% *v*/*v*. After 1 hr, indicator Rh-positive red cells opsonized with anti-RhD were added to the chamber slides and then further incubated for 2 hrs before fixing and counting phagocytosed RBCs using phase-contrast microscopy [7]. As a positive control for inhibition, IVIG, known to inhibit phagocytosis due to blocking of FcγRs, was used [7]. The phagocytosis index (PI) was determined as the number of phagocytosed RBCs in 100 monocytes. The percent inhibition of phagocytosis was determined by comparing the PI in each treated sample to the PI in the untreated sample, which was determined by normalizing the PI concentration in each treated sample (phagocytosis of opsonized RBCs with vehicle only, representing 100 percent phagocytosis).

(1) $$\mathrm{PI}=\frac{\#\;\mathrm{of}\;\mathrm{phagocytic}\;\mathrm{RBCs}}{300\;\mathrm{monocytes}}\times 100$$

### 4.8. Cell Viability Assays

MTT Assay. The MTT assay was used to assess the impact of the selected compounds on the metabolic activity of primary mononuclear cells [12,13]. PBMCs (5 × 104 total cells per well) were isolated from ACD tubes and seeded on 96 culture plates (VWR^®^ Tissue Culture Plate, Untreated, Sterilized, Non-Pyrogenic). Cells were treated with 5 µM of the solubilized compounds and incubated for three hours at 37 °C with 5% CO_2_. After that, MTT was added to the cells and plates were incubated for 2 hrs. The formation of formazan purple crystals was followed under a light microscope. We solubilized the formazan crystals by incubating overnight with 100 µL of 10% SDS, 0.01M HCl SDS. Absorbance was measured at 570 (reference 690 nm) in a BioTek EPOCH 2 microplate reader with Gen 5 Ver. 2.05 software (BioTek, Winooski, VT, USA). As a positive control for decreased metabolic activity, 100 µM thimerosal (diluted in RPMI) was used. Untreated cells represented the basal metabolic activity of the cells. This experiment is repeated at increasing concentrations (250 µM, 100 µM, 50 µM, and 10 µM) for the two lead compounds (KB-151 and KB-208) using a variety of cell types, including PBMC, Hep G2, and HEK-293.

LDH Assay. To understand if any of the studied compounds induced primary cell death (PBMC), LDH release was evaluated as a measure of cytotoxicity [10]. To this end, an LDH-cytotoxicity test kit (Sigma Inc., Marlborough, MA, USA) was used according to the manufacturer’s instructions. Sample treatment was performed identically to the MTT assay previously described. The absorbance at 490 and 600 nm was determined using a microplate reader (Bio-Rad EPOCH II). Minimum lysis control is referred to as samples without treatment. Cells solubilized with a lysis buffer provided with the kit are considered as maximum lysis control. Specific cell death was calculated as:

(2) $$\%\;\mathrm{Specific}\;\mathrm{Death}=\frac{\left(\mathrm{Experimental}\;\mathrm{Lysis}-\mathrm{Minimum}\;\mathrm{Lysis}\right)}{\left(\mathrm{Maximum}\;\mathrm{Lysis}-\;\mathrm{Minimum}\;\mathrm{Lysis}\right)}\times 100$$

This experiment was repeated at increasing concentrations (250 µM, 100 µM, 50 µM, and 10 µM) for the two lead compounds (**KB-151** and **KB-208**) using a variety of cell types, including PBMC, Hep G2, and HEK-293.

### 4.9. Apoptosis Assay

PBMC, Hep G2, and HEK-293 cells were treated for 1 hr with KB-151 and **KB-208** at a 250 µM concentration. Thimerosal (100 µM) was utilized as a positive control for viability (diluted in RPMI). Staining with Annexin V and PI was performed according to the manufacturer’s procedure for the Annexin-V-FLUOS Staining Kit (Sigma), and samples were run through an SP6800 Spectral Cytometer.

### 4.10. Dose-Inhibitory Response and IC_50_

MMAs (as explained in detail in Section 4.7) were repeated with different concentration of four hit compounds (**KB-151**, **KB-198**, **KB-208**, and **KB-210**), including 100 µM, 50 µM, 25 µM, 10 µM, 5 µM, 2.5 µM, 1 µM, and 0.5 µM with human PBMCs. Dose-inhibitory response experiments were also repeated for RAW 264.7 and mouse (BA:B/c) PBMCs with different concentrations of two lead compounds (**KB-151** and **KB-208**), including 400 µM, 200 µM, 100 µM, 50 µM, 25 µM, and 10µM.

## Figures and Tables

**Table 1:** Compounds that inhibited anti-D-opsonized Rh(D+) by ≥ 40% phagocytic inhibition.

Chemical Compound	PI ^1^-Average	Inhibition%
KB-151	17	80
KB-167	14	53
KB-175	15	50
KB-178	29	53
KB-179	14	53
KB-181	37	45
KB-182	28	55
KB-186	28	55
KB-189	30	52
KB-190	27	56
KB-198	9	75
KB-199	40	40
KB-205	19	47
KB-206	18	50
KB-208	7	81
KB-209	18	50
KB-210	11	69
KB-212	19	47
KB-213	13	64

^1^ Phagocytosis Index $\left(\mathrm{PI}\right)=\frac{\#\;\mathrm{of}\;\mathrm{phagocytic}\;\mathrm{RBCs}}{300\;\mathrm{monocytes}}\times 100$.

## Data Availability

Not applicable.

## Supplementary Materials

The following supporting information can be downloaded at: https://www.mdpi.com/article/10.3390/molecules28020757/s1, Figure S1: Unpublished 3rd generation structures based on merging the features of **KB-55**, **KB-57,** and **KB-59** [6]; Figure S2: ^1^H-NMR spectrum of **KB-151** in CDCl_3_; Figure S3: ^1^H-NMR spectrum of **KB-198** in CDCl_3_; Figure S4: ^1^H-NMR spectrum of **KB-208** in CDCl_3_; Figure S5: ^1^H-NMR spectrum of **KB-210** in CDCl_3_; Figure S6: UHPLC-HRMS/MS data of **KB-151**; Figure S7: UHPLC-HRMS/MS data of **KB-198**; Figure S8: UHPLC-HRMS/MS data of **KB-208**; Figure S9: UHPLC-HRMS/MS data of **KB-210**; Figure S10: HPLC-PDA chromatogram of **KB-151**; Figure S11: HPLC-PDA spectrum of **KB-151**; Figure S12: HPLC-PDA chromatogram of **KB-198**; Figure S13: HPLC-PDA spectrum of **KB-198**; Figure S14: HPLC-PDA chromatogram of **KB-208**; Figure S15: HPLC-PDA spectrum of **KB-208**; Figure S16: HPLC-PDA chromatogram of **KB-210**; Figure S17: HPLC-PDA spectrum of **KB-210**; Table S1: Purity results of hit compounds.
