# Supplementary material for: Small Molecule Drugs That Inhibit Phagocytosis
Source: Molecules. 2023 Jan 12;28(2):757. doi: 10.3390/molecules28020757 (PMC9867408; doi:10.3390/molecules28020757)
Supplement: Supplementary file 1 [file molecules-28-00757-s001.zip › molecules-2147530-supplementary.pdf]

Supplementary Information
Small Molecule Drugs That Inhibit Phagocytosis

Melika Loriamini [1,2], Melissa M. Lewis-Bakker [3], Kayluz Frias Boligan [1], Siming Wang [3], Mairead B. Holton [1], Lakshmi P. Kotra [3,4,*] and Donald R. Branch [1,2,5,6,*]

[1] Centre for Innovation, Canadian Blood Services, Toronto, ON M5G 2M1, Canada
[2] Department of Laboratory Medicine and Pathobiology, University of Toronto, Toronto, ON M5S 1A8, Canada
[3] Krembil Research Institute, University Health Network, Toronto, ON M5G 1L7, Canada
[4] Department of Pharmaceutical Sciences, Leslie Dan Faculty of Pharmacy, University of Toronto, Toronto, ON M5S 3M2, Canada
[5] Department of Medicine, University of Toronto, Toronto, ON M5S 1A8, Canada
[6] Keenan Research Centre, Canadian Blood Services, Toronto, ON M5B 1W8, Canada
*Correspondence: p.kotra@utoronto.ca (L.P.K.); don.branch@utoronto.ca (D.R.B.);
Tel.: +1-416-581-7602 (L.P.K.); +1-416-313-4458 (D.R.B.)

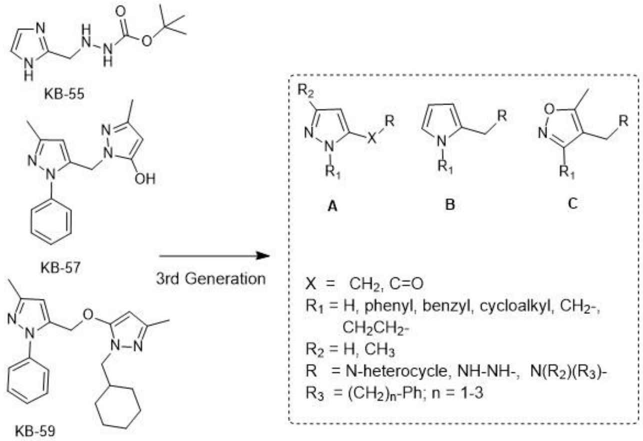

Figure S1. Unpublished 3rd generation structures based on merging the features of **KB-55**, **KB-57** [6] and **KB-59** [6].

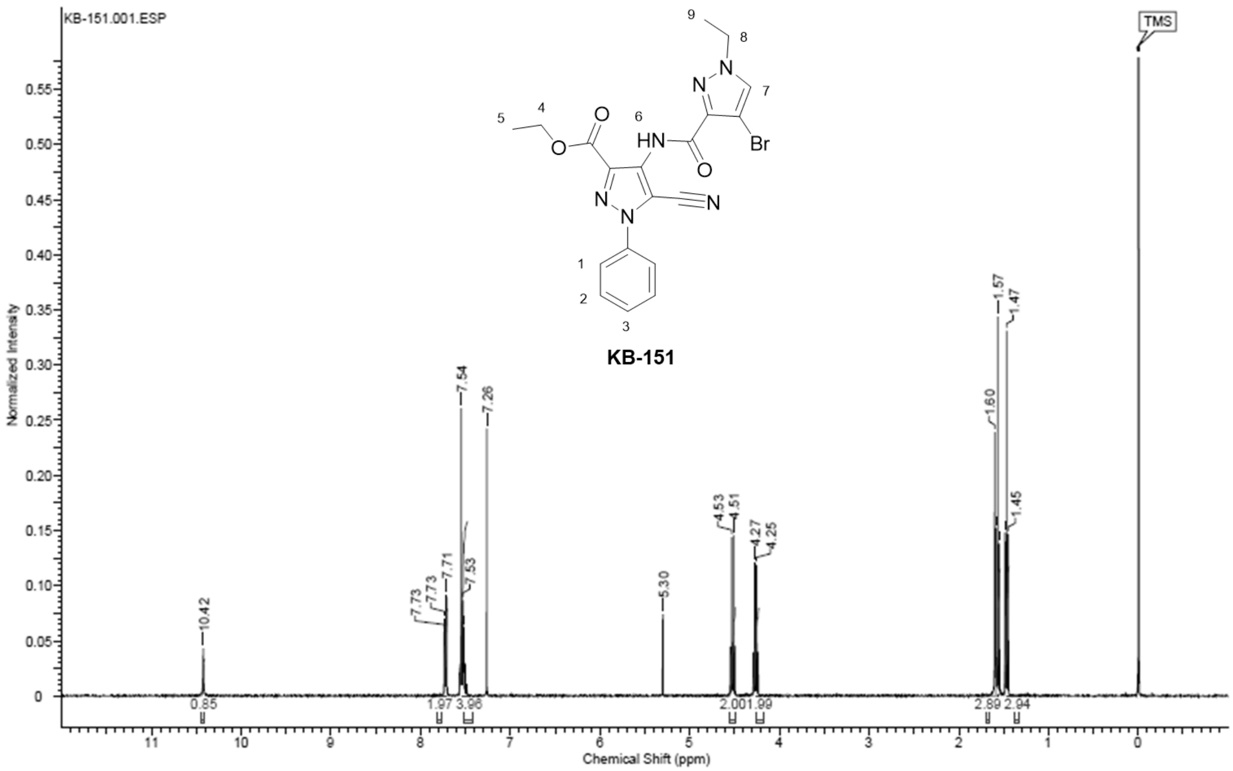

Figure S2. ^{1}H-NMR spectrum of **KB-151** obtained using the method as described in Section 4.2 of main text.

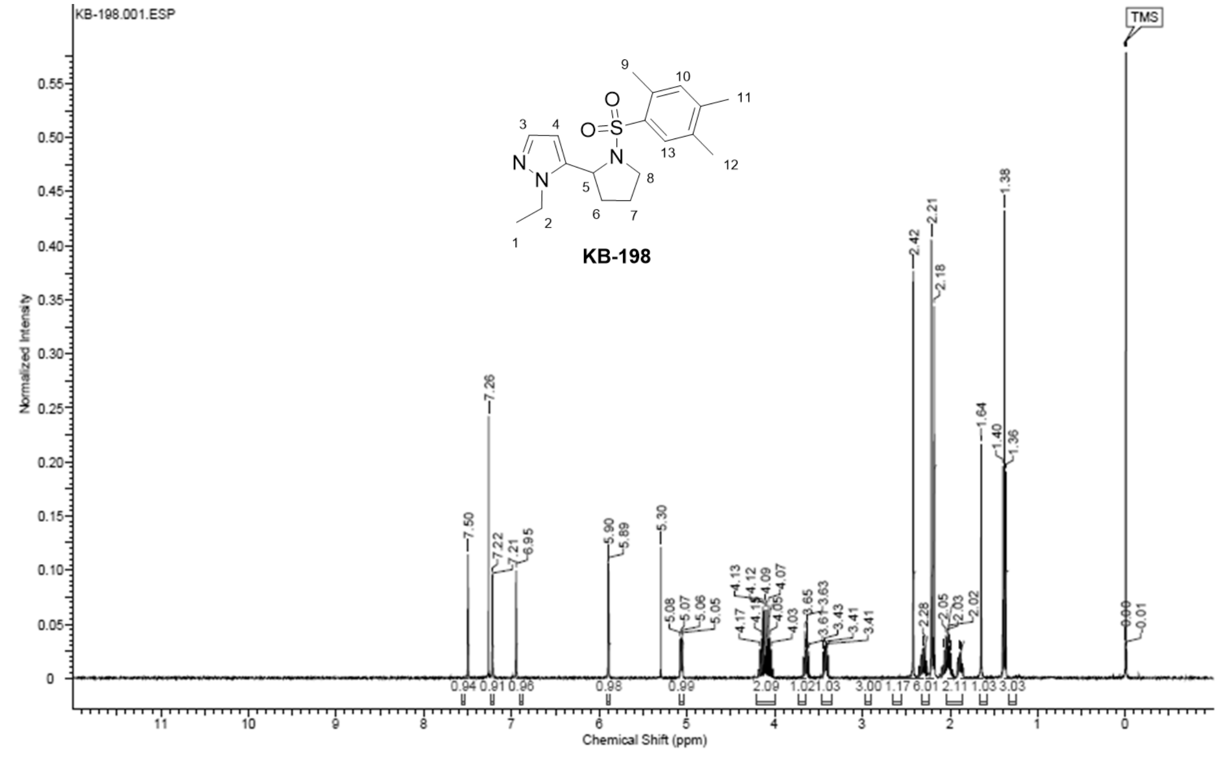

Figure S3. ^{1}H-NMR spectrum of **KB-198** obtained using the method as described in Section 4.2 of main text.

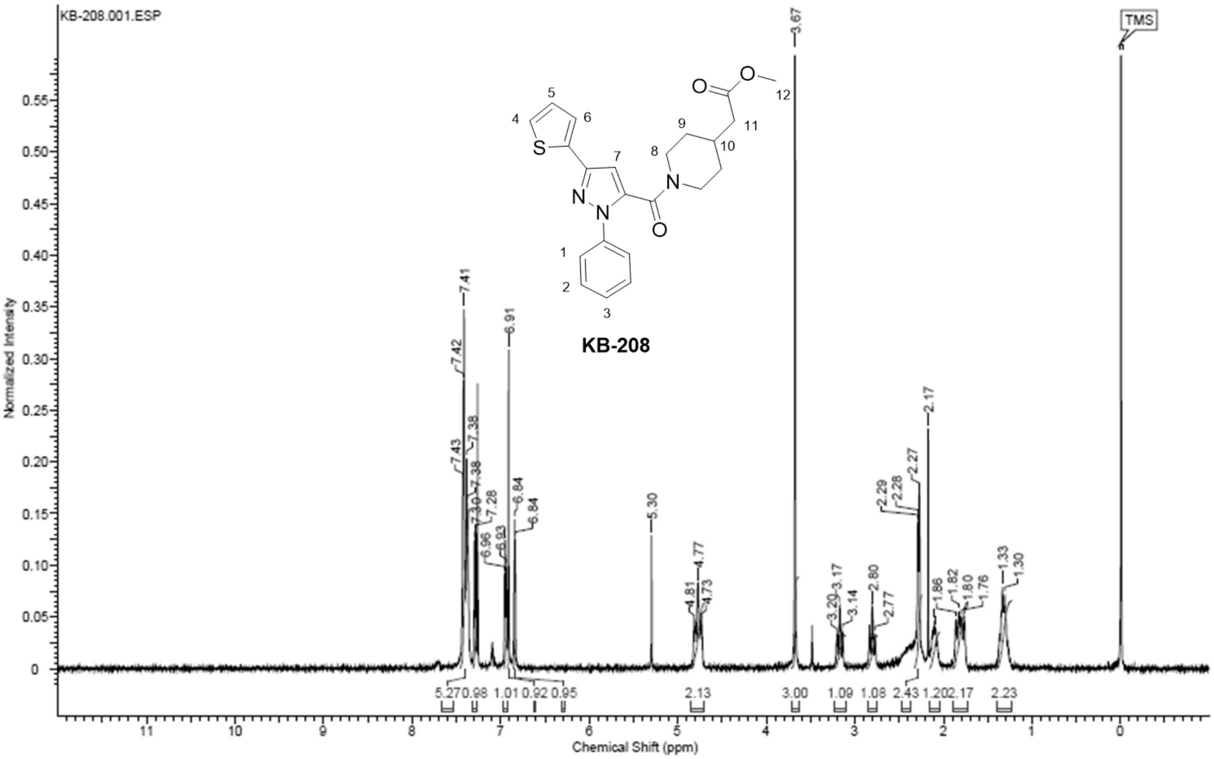

Figure S4. ¹H-NMR spectrum of **KB-208** obtained using the method as described in Section 4.2 of main text.

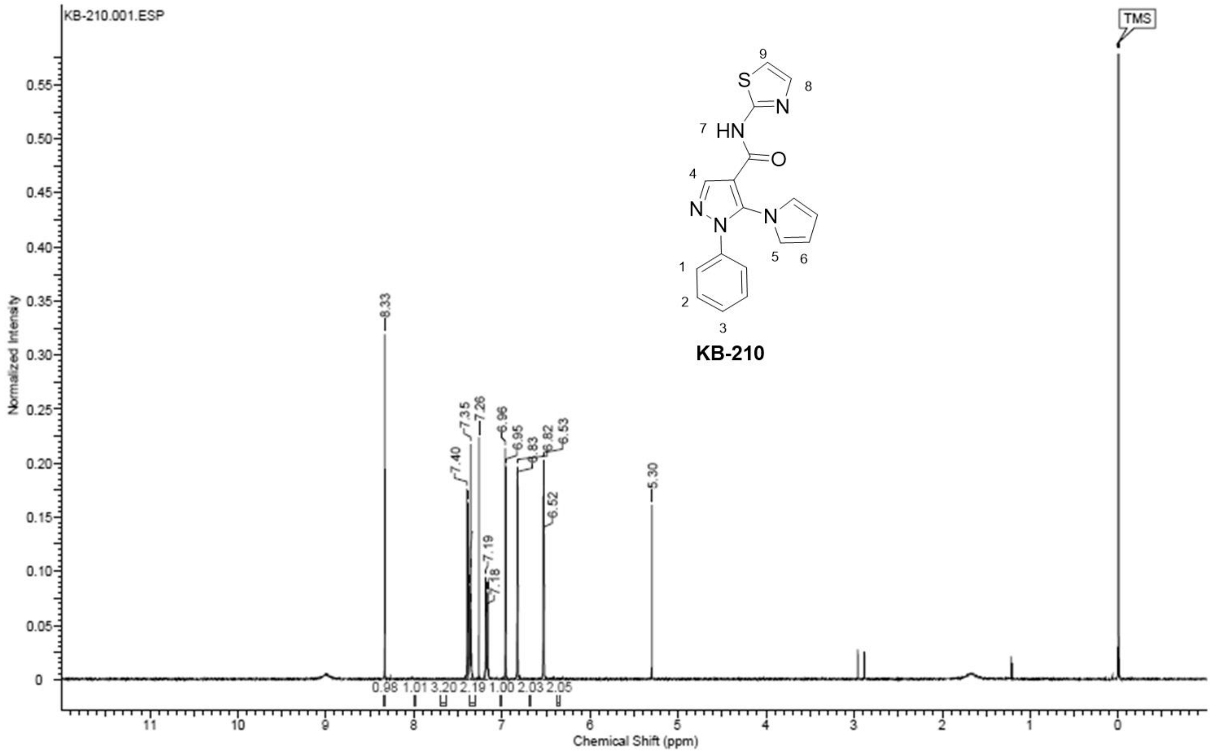

Figure S5. ¹H-NMR spectrum of **KB-210** obtained using the method as described in Section 4.2 of main text.

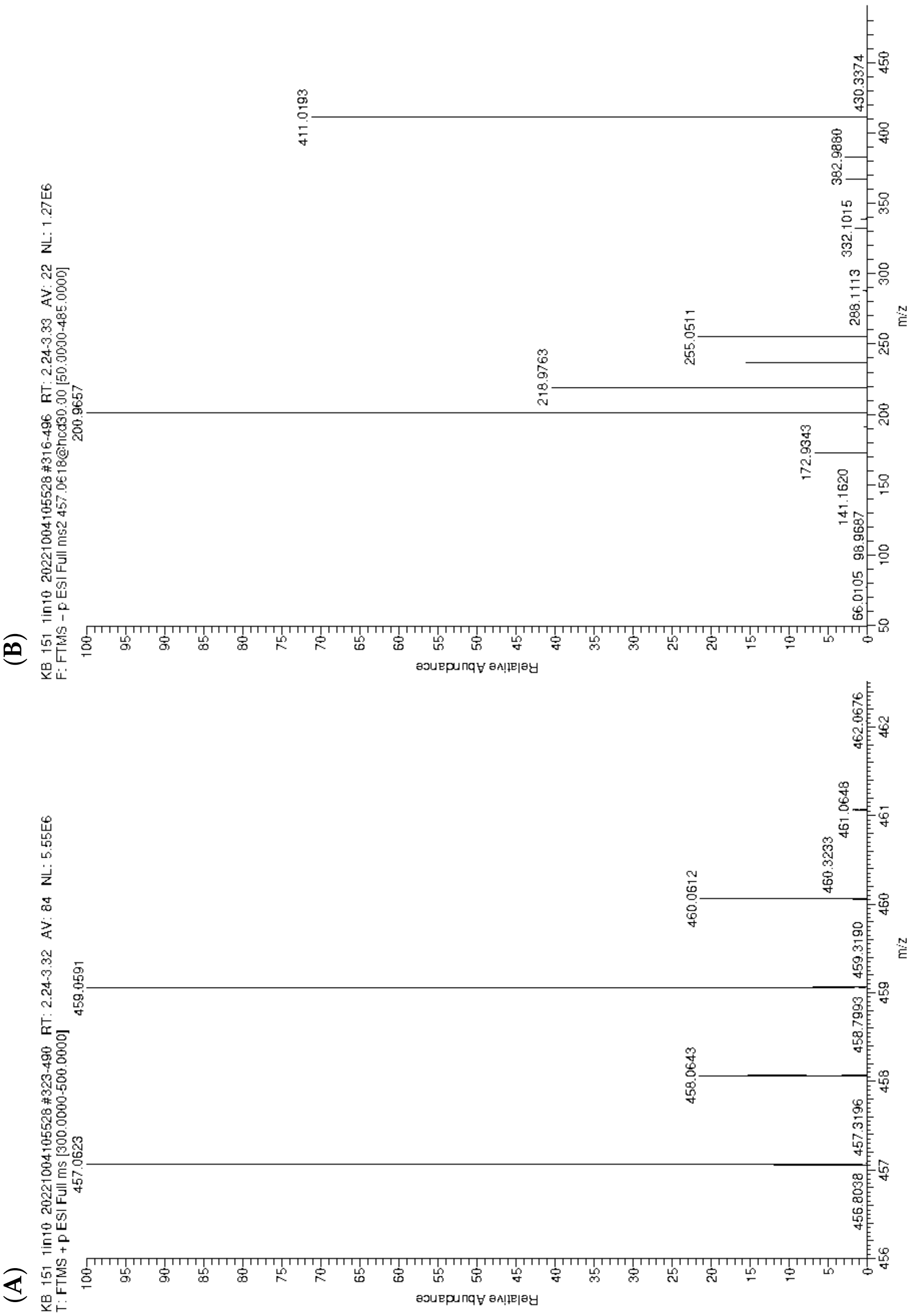

Figure S6. UHPLC-HRMS/MS spectrum of **KB-151** obtained using the method as described in Section 4.3 of main text. (**A**) MS1 and (**B**) MS2.

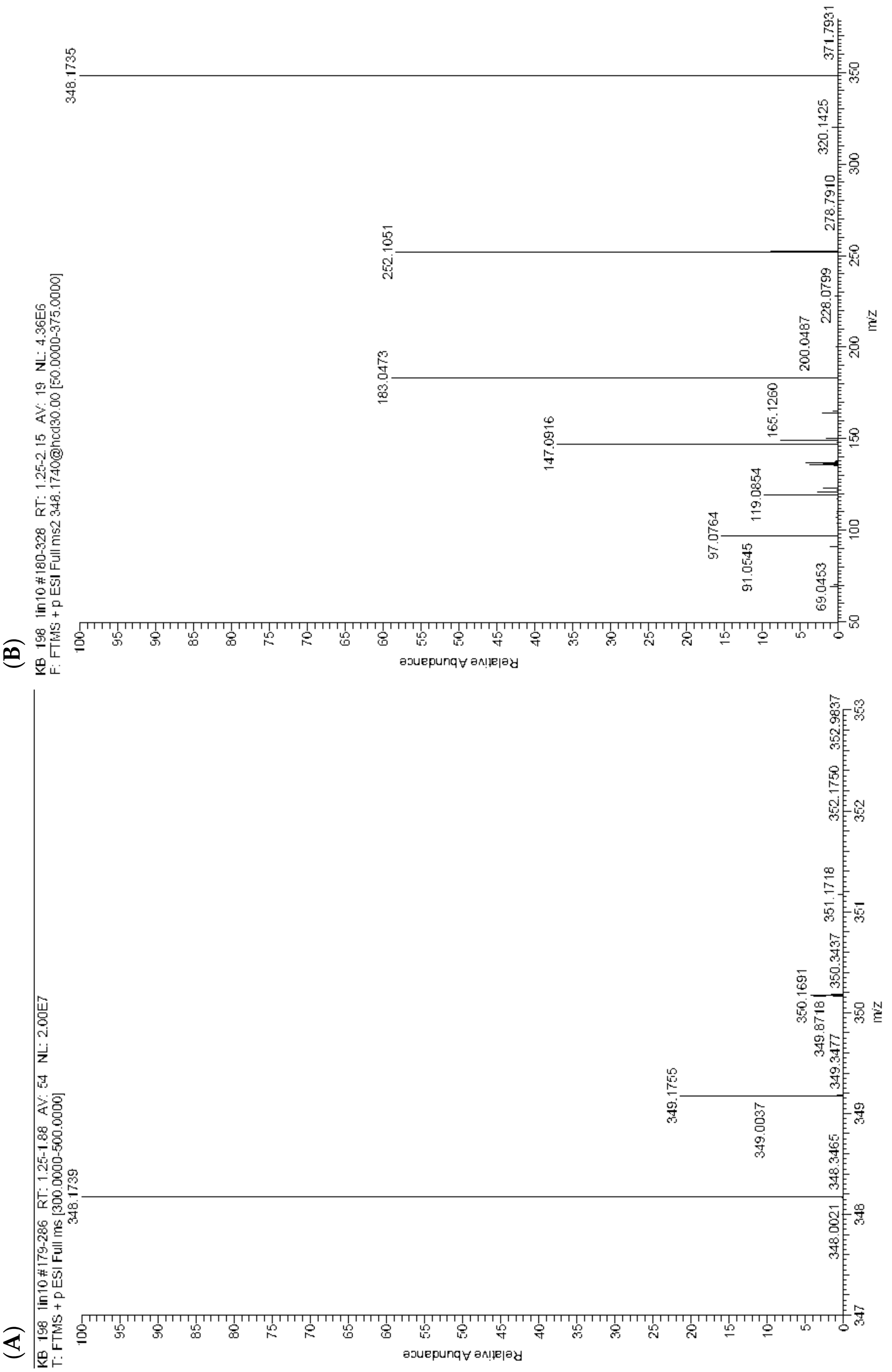

Figure S7. UHPLC-HRMS/MS spectrum of **KB-198** obtained using the method as described in Section 4.3 of main text. (**A**) MS1 and (**B**) MS2.

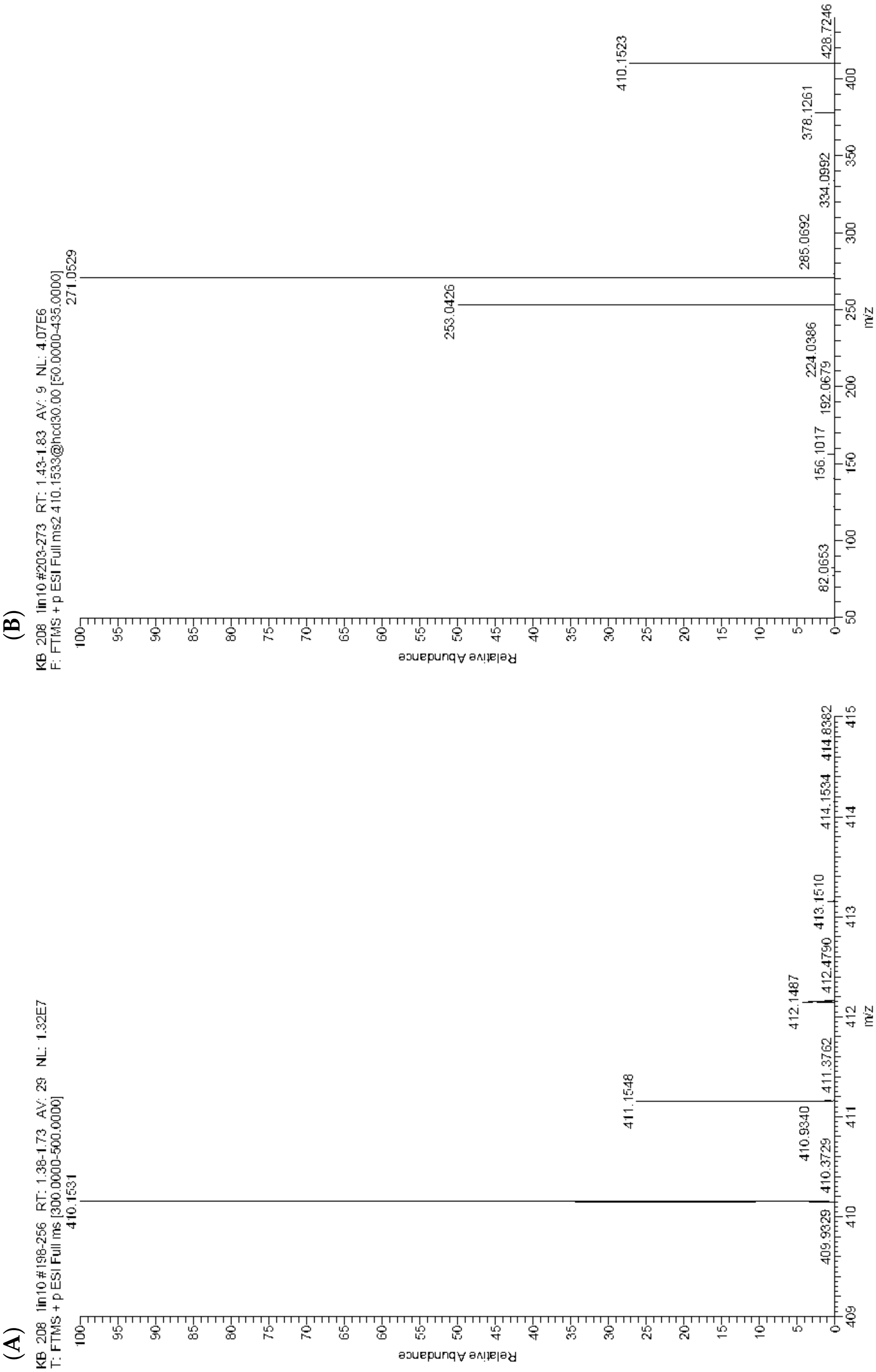

Figure S8. UHPLC-HRMS/MS spectrum of **KB-208** obtained using the method as described in Section 4.3 of main text. (**A**) MS1 and (**B**) MS2.

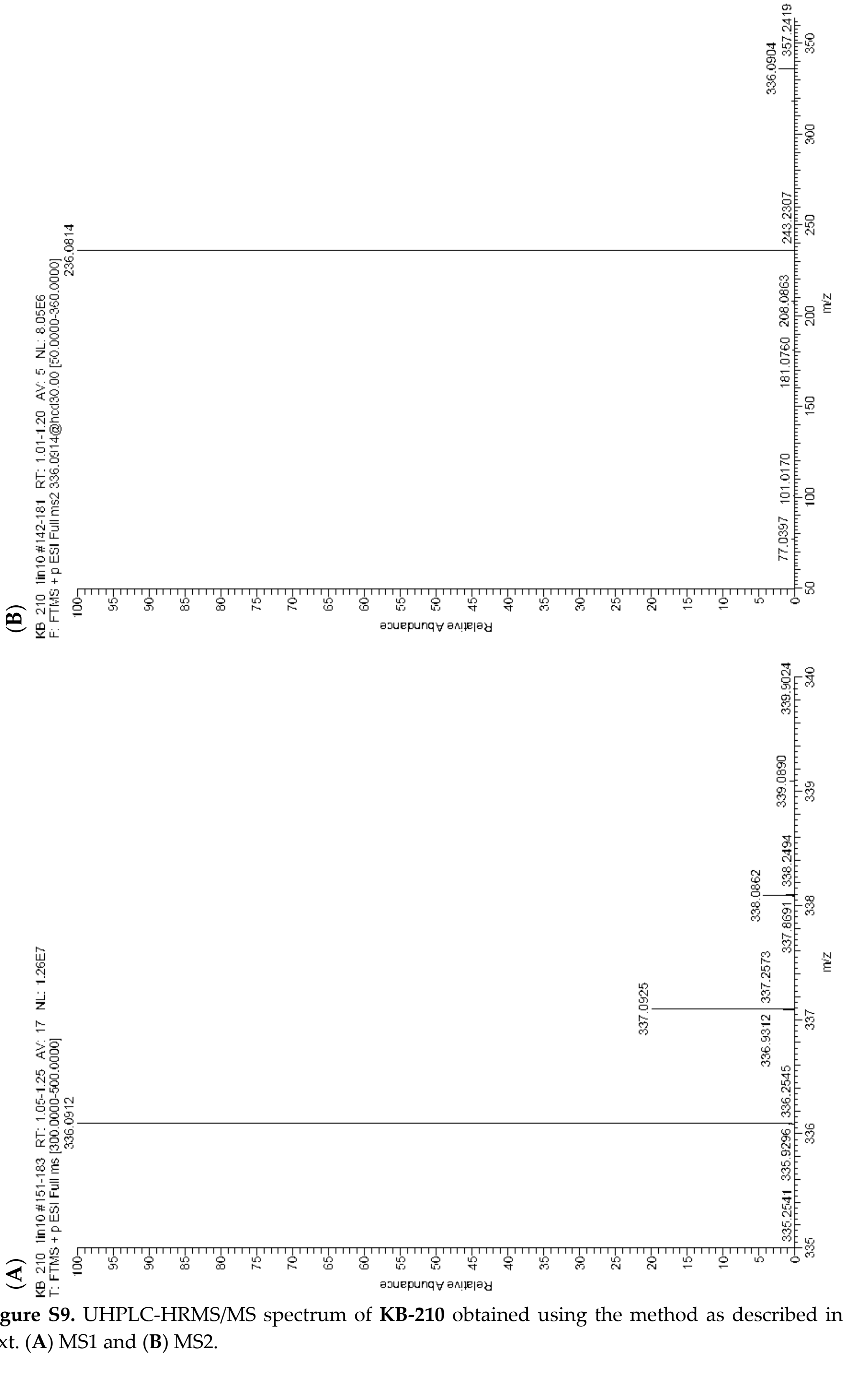

Figure S9. UHPLC-HRMS/MS spectrum of **KB-210** obtained using the method as described in Section 4.3 of main text. (**A**) MS1 and (**B**) MS2.

Table S1. Purity results of hit compounds using methods as described in Section 4.4 of main text.

**Chemical Compound**	**Purity (%)**	
	**Method A**	**Method B**
**KB-151**	95	97
**KB-198**	98	96
**KB-208**	95	95
**KB-210**	99	97

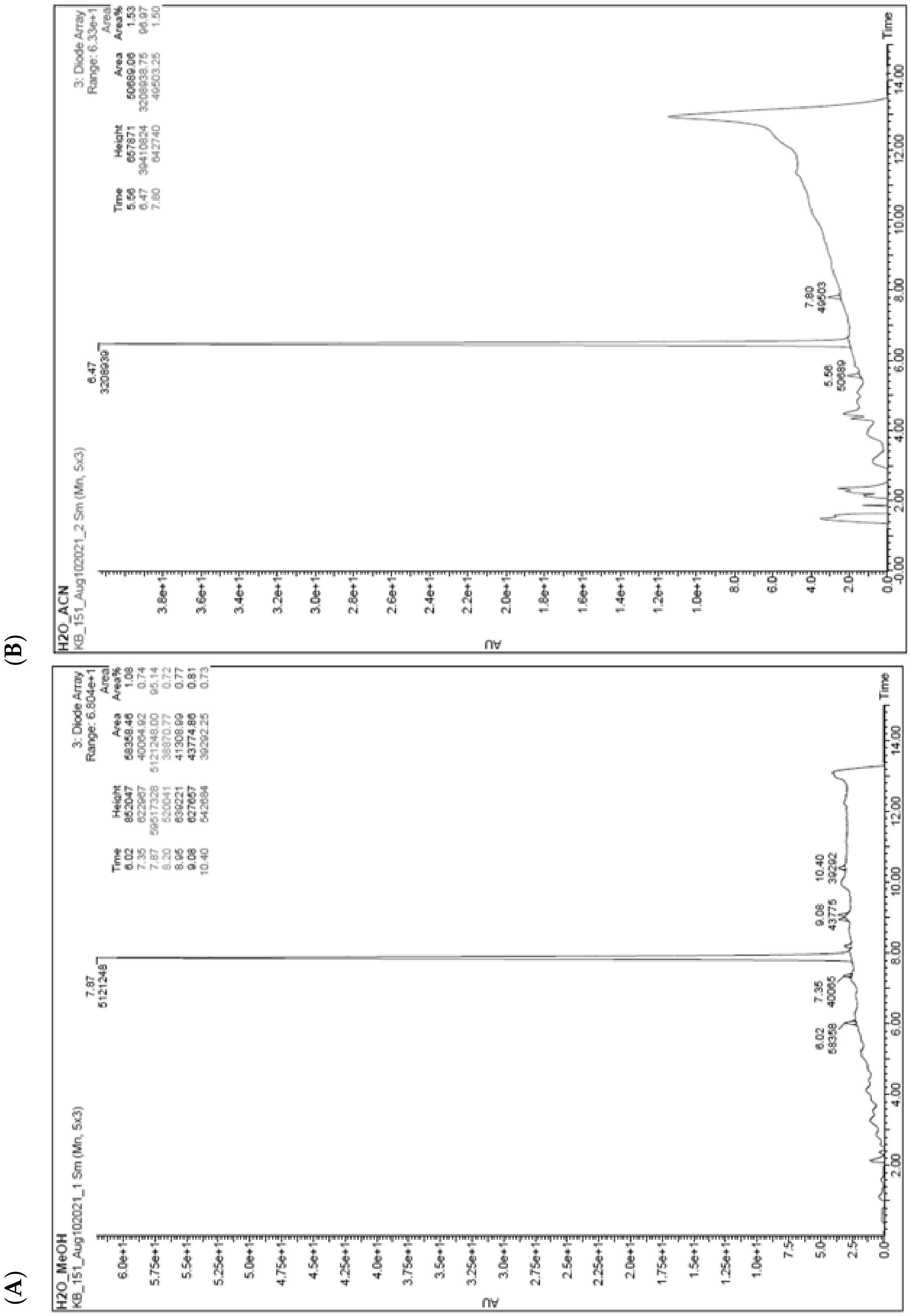

Figure S10. HPLC-PDA chromatogram of **KB-151** using the method as described in Section 4.4 of main text. (**A**) Method A and (**B**) Method B.

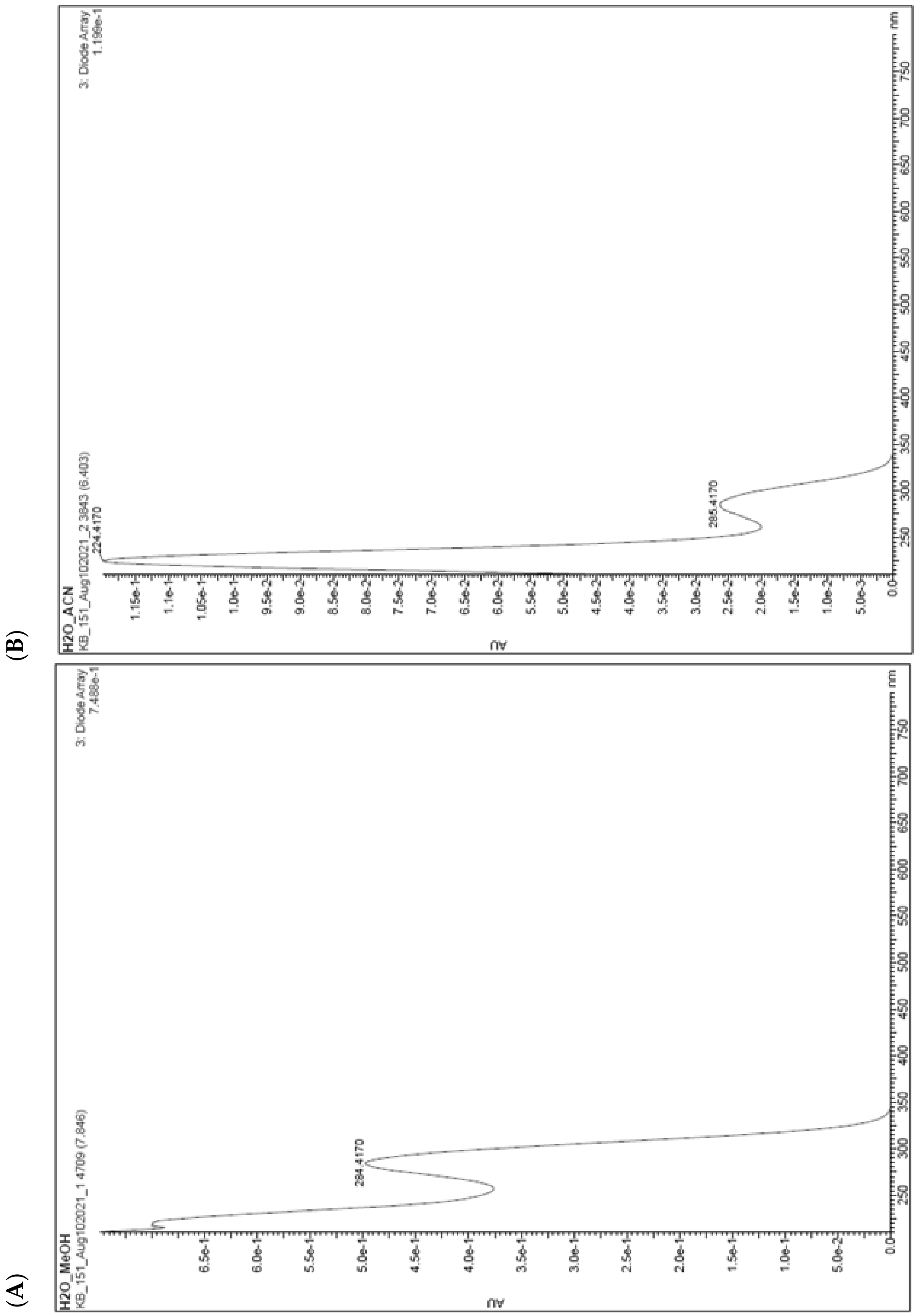

Figure S11. HPLC-PDA spectrum of **KB-151** using the method as described in Section 4.4 of main text. (**A**) Method A and (**B**) Method B.

(A) (B)

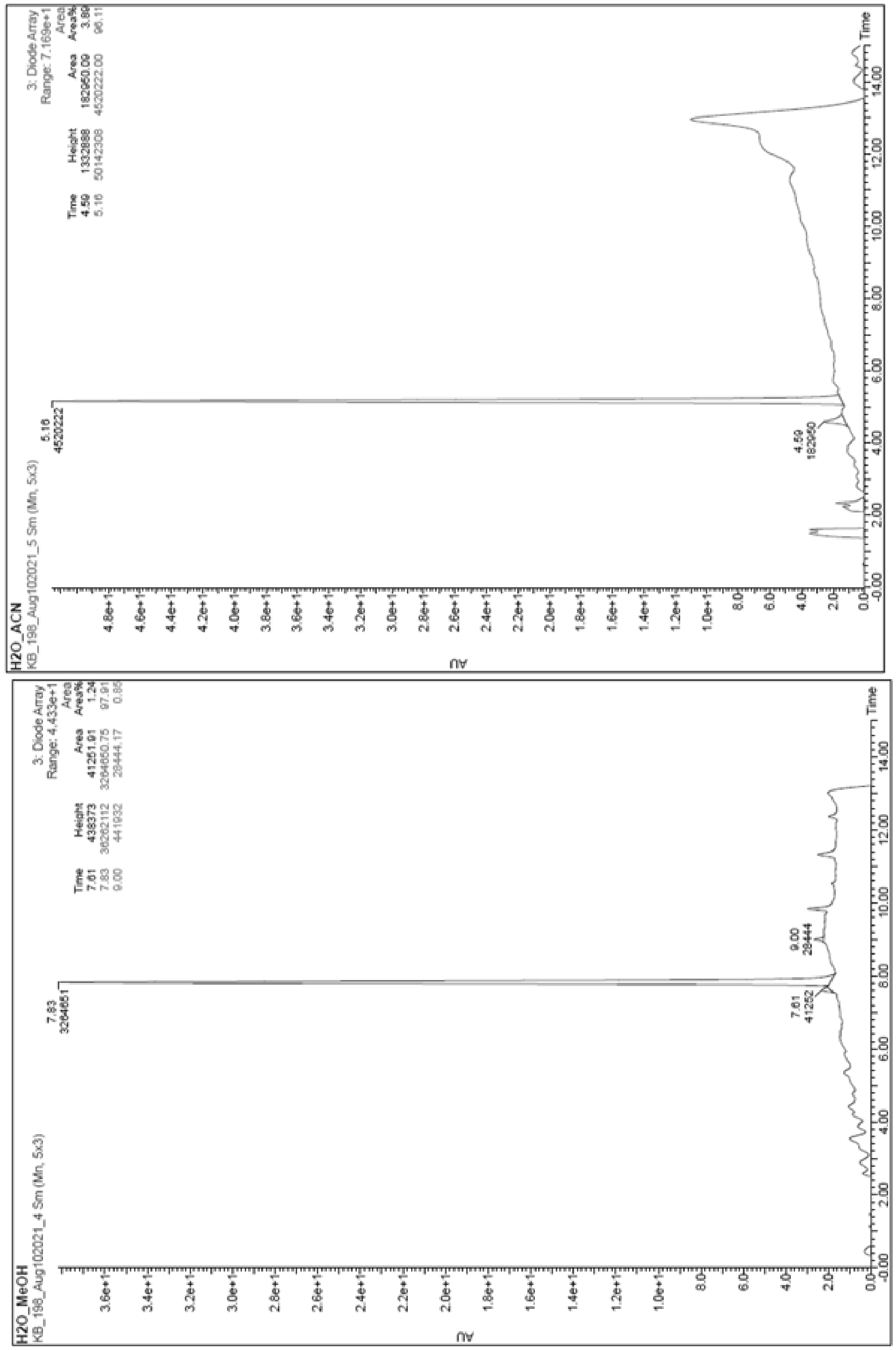

Figure S12. HPLC-PDA chromatogram of **KB-198** using the method as described in Section 4.4 of main text. (**A**) Method A and (**B**) Method B.

(B)

(A)

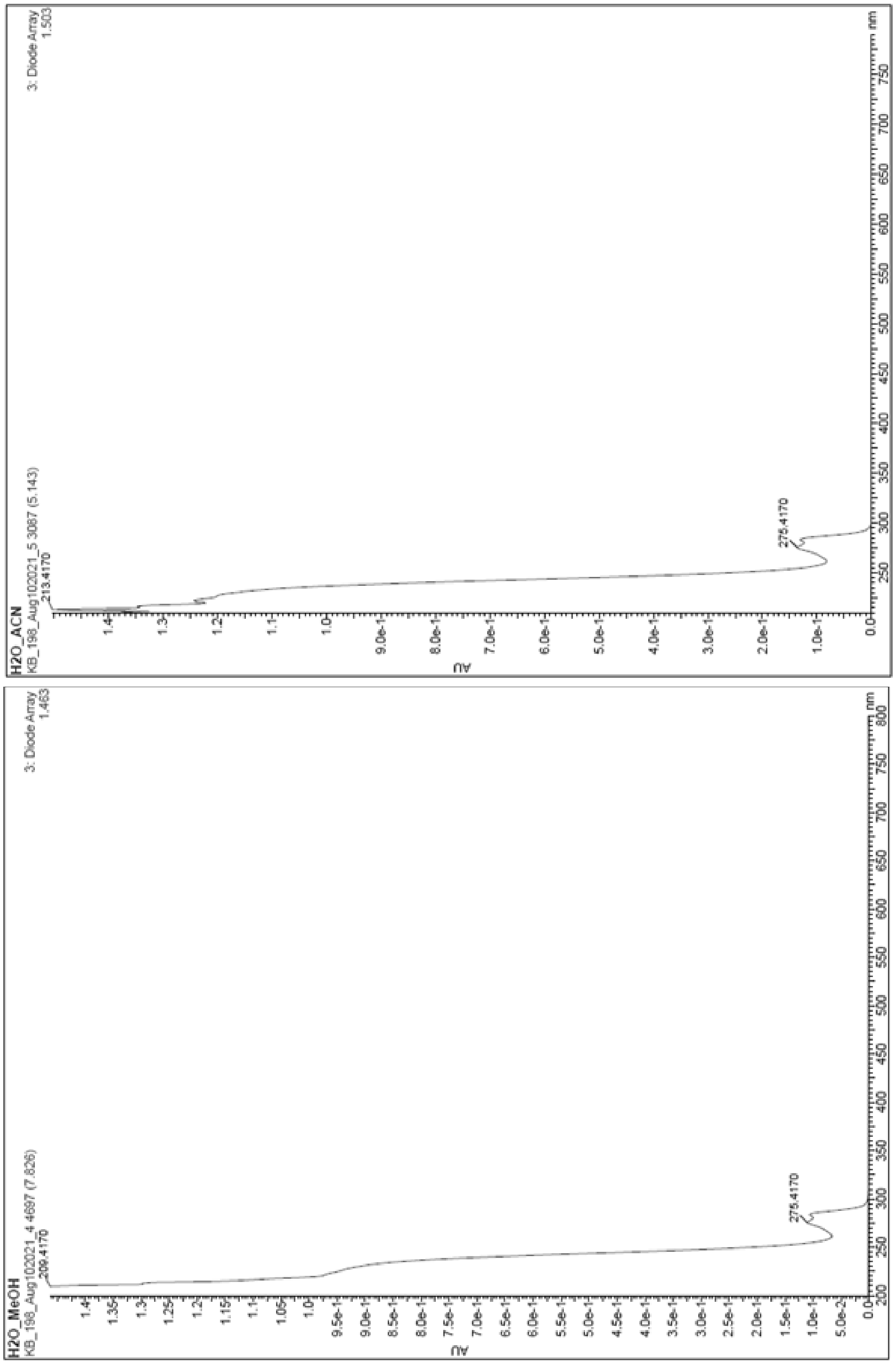

Figure S13. HPLC-PDA spectrum of **KB-198** using the method as described in Section 4.4 of main text. (**A**) Method A and (**B**) Method B.

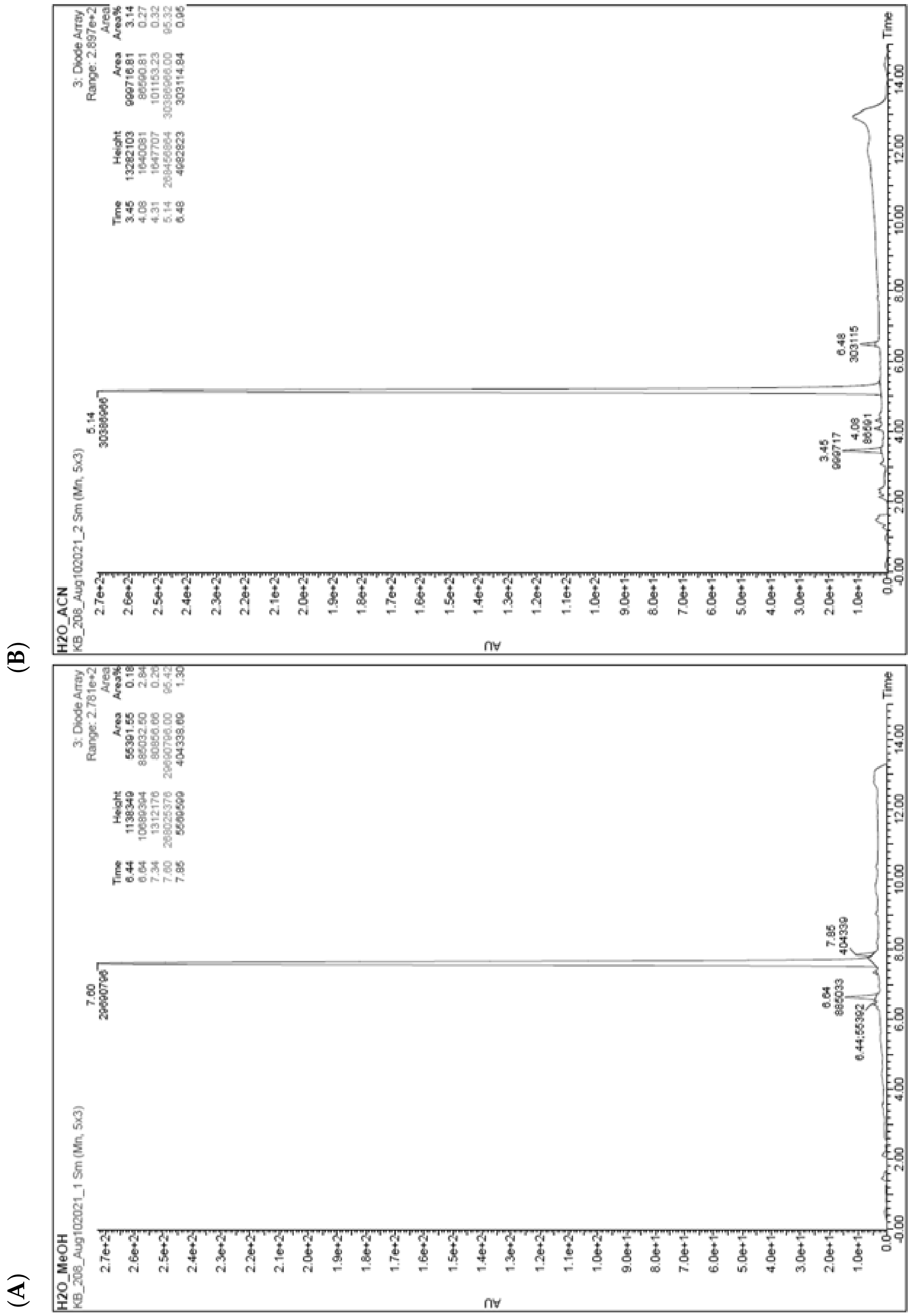

Figure S14. HPLC-PDA chromatogram of **KB-208** using the method as described in Section 4.4 of main text. (**A**) Method A and (**B**) Method B.

(A) (B)

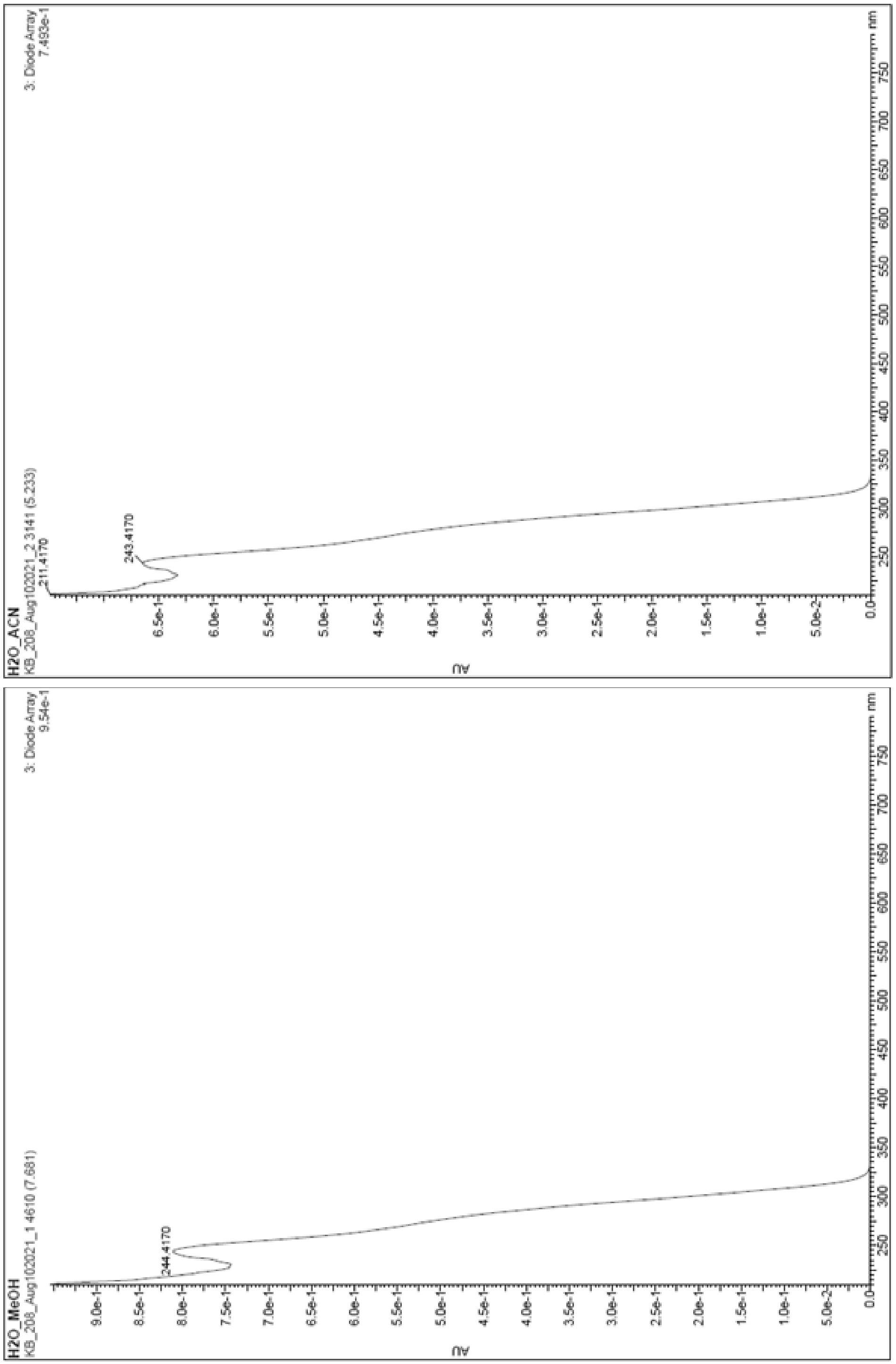

Figure S15. HPLC-PDA spectrum of **KB-208** using the method as described in Section 4.4 of main text. (**A**) Method A and (**B**) Method B.

(A) (B)

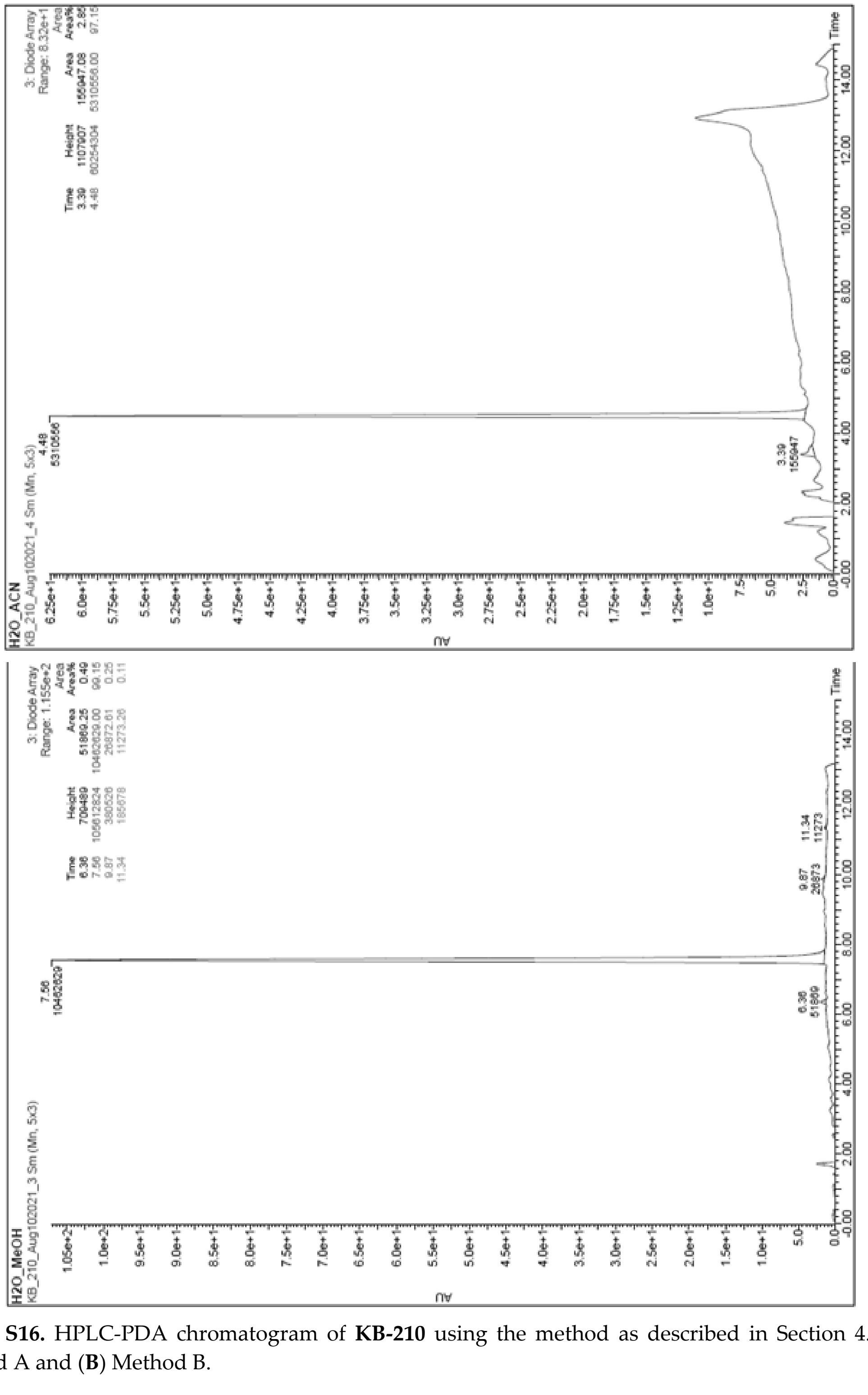

Figure S16. HPLC-PDA chromatogram of **KB-210** using the method as described in Section 4.4 of main text. (**A**) Method A and (**B**) Method B.

(A) (B)

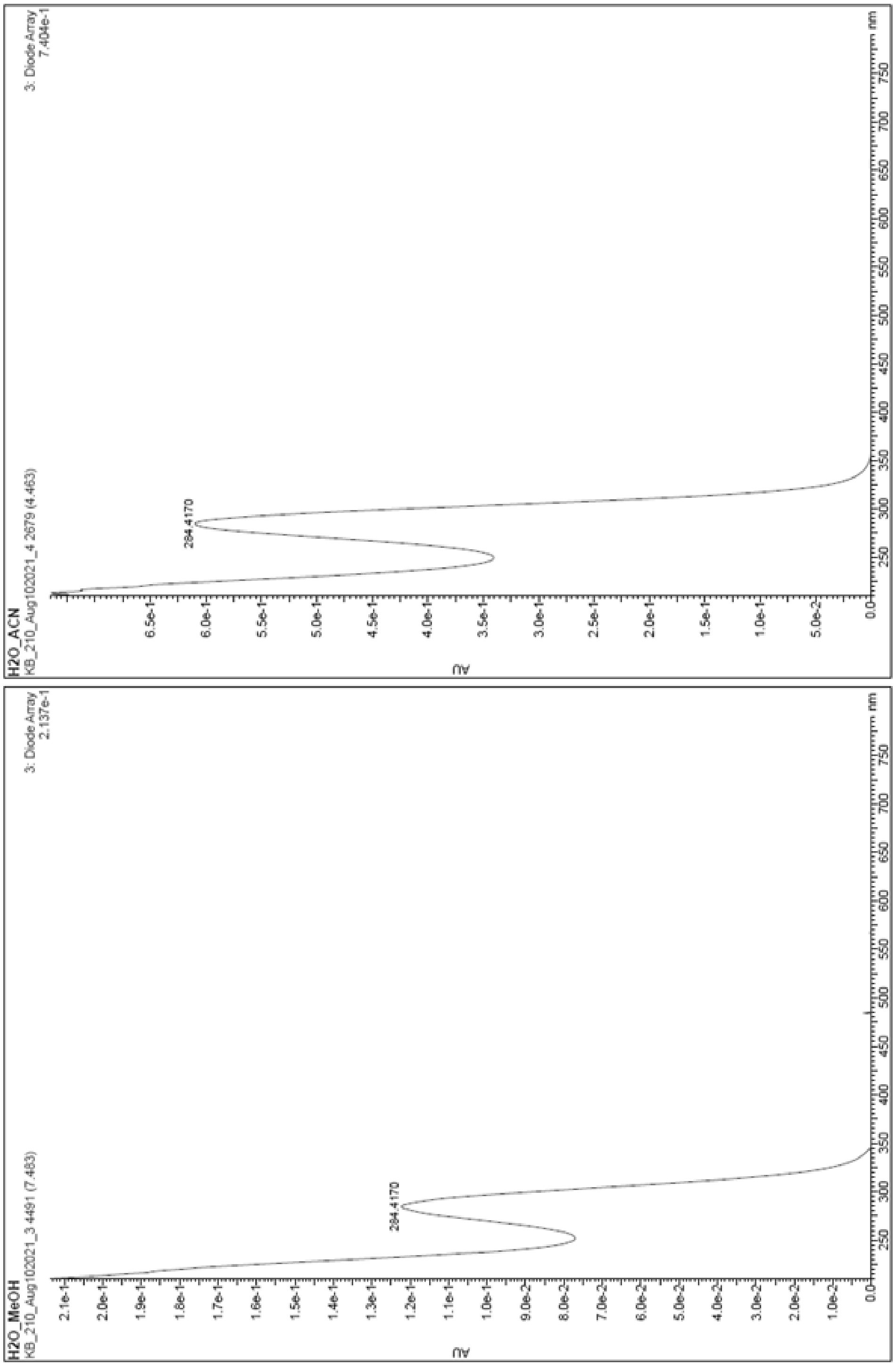

Figure S17. HPLC-PDA spectrum of **KB-210** using the method as described in Section 4.4 of main text. (**A**) Method A and (**B**) Method B.

Reference

6. Purohit, M.K.; Chakka, S.K.; Scovell, I.; Neschadim, A.; Bello, A.M.; Salum, N.; Katsman, Y.; Bareau, M.C.; Branch, D.R.; Kotra, L.P. Structure-activity relationships of pyrazole derivatives as potential therapeutics for immune thrombocytopenias. *Bioorg. Med. Chem.* **2014**, *22*, 2739–2752. https://doi.org/10.1016/j.bmc.2014.03.016.
